# Chimera: An atlas of regular vines on up to 8 nodes

**DOI:** 10.1038/s41597-023-02252-6

**Published:** 2023-05-31

**Authors:** Oswaldo Morales-Nápoles, Mojtaba Rajabi-Bahaabadi, Gina Alexandra Torres-Alves, Cornelis Marcel Pieter ’t Hart

**Affiliations:** 1 Delft University of Technology, Faculty of Civil Engineering and Geosciences, Delft, 2628CN The Netherlands; 2 Yazd University, Civil Engineering, Yazd, 8915818411 Iran; 3 Tunnel Engineering Consultants, Amersfoort, The Netherlands

**Keywords:** Statistics, Applied mathematics, Computer science

## Abstract

Vine copulas have become the standard tool for modelling complex probabilistic dependence. It has been shown that the number of regular vines grows extremely quickly with the number of nodes. Chimera is the first attempt to map the vast space of regular vines. Software for operating with regular vines is available for R, matlab and Python. However, no dataset containing all regular vines is available. Our atlas of regular vines, *Chimera*, comprises all 24 4 × 4 matrices representing regular vines on 4 nodes, 480 5 × 5 matrices representing regular vines on 5 nodes, 23,040 6 × 6 matrices representing regular vines on 6 nodes, 2,580,480 7 × 7 matrices representing regular vines on 7 nodes and 660,602,880 8 × 8 matrices representing regular vines on 8 nodes. Regular vines in Chimera are classified according to their tree-equivalence class. We fit all regular vines to synthetic data to demonstrate the potential of Chimera. Chimera provides thus a tool for researchers to navigate this vast space in an orderly fashion.

## Background & Summary

Regular vines are graphs (or a sequence of graphs) that facilitate the characterization of complex multidimensional probability distributions. Regular vines used together with bivariate copulas, are the building blocks of multivariate distributions commonly referred to as vine copulas. The first vine copula and non explicitly also the first regular vine was introduced by Joe in 1994^1^ while the first formal definition of regular vines (and vine copulas) was presented by Cooke in 1997^2^. Only in 2009, were vine copulas presented as statistical models^3^. Their flexibility has made them become the standard tool for modelling complex multidimensional probability distributions in different fields. Vine copulas add flexibility because they construct a probability distribution from bi-variate pieces rather than trying to represent a joint distribution with a particular multidimensional parametric family.

While theoretical developments are still being made, vine copulas on a different number of variables have found application in virtually all fields of science and engineering. Recent example applications can be found in finance, business and economics^4–10^, coastal management^11^, earth sciences^12–14^ and engineering^15–23^, where the number of variables in their respective vine copula models ranges from 3 to 10 variables. In a recent study by the authors, vine copulas on 6 variables (23,040 models) are fit to two sets of variables including waves, currents and hydrodynamic forces acting on a submerged floating tunnel for its evaluation under different design configurations. In health sciences, the spatial dependence for COVID-19 infection rates was modeled with a vine copula of 21 variables^24^, while a vine copula of 4 variables was implemented to create a secure method to transfer sensitive data without accidental leakages^25^.

Despite their popularity for modelling multidimensional probability distributions, the use of vine copulas on 6 or more variables relies mostly on heuristics^26^. This is partly because the non-unique decomposition of the multidimensional probability distributions in bi-variate building blocks causes the number of regular vines to grow extremely quickly with the number of variables under consideration. In particular, previous research has shown that the number of regular vines on *d* nodes is $$\frac{d!}{2}\times {2}^{\left(\begin{array}{c}d-2\\ 2\end{array}\right)}$$^27,28^. Notice that this number for 4 to 8 nodes corresponds already to 663,206,904 regular vines. The heuristics previously mentioned have been poorly tested, to some extent because a dataset containing all regular vines on more than 5 nodes is not available. In fact, an atlas of regular vines in higher dimension would enable brute force testing of all possible regular vine structures (assuming unlimited computational power) paving the way to improved heuristics. Regular vines in 5 nodes have been obtained in the past though permutation per equivalence classes (see for example^29^). To our knowledge, this method has not been successfully used for more than 5 variables, neither a dataset with regular vine matrices on more than 5 variables is available.

In order to fill this gap, in this paper we introduce our atlas of regular vines from 4 to 8 elements: Chimera. A Chimera is an imaginary creature from Greek mythology that has the head of a lion, mid body of a goat and lower body of a serpent. Like all fantastic creatures, it is made up of “simpler” pieces of other real or imaginary creatures. Trees are the “simpler” pieces that give rise to vines. Regular vines are very much created like the zoology of the fantastic. In order to remind us of this fact our atlas is named Chimera. The data contained in Chimera consists of 663,206,904 matrices representing the regular vines of interest. The objective of this paper is thus to make these matrices available to researchers rather than providing new algorithms for producing them or a new proof of the number of regular vines as a function of the number of nodes. The data is available for R, matlab and Python since software implementations for manipulating vine copulas exist in all 3 languages^30–32^. Finally, we illustrate the potential of Chimera by fitting all vine copulas from 4 to 8 nodes to synthetic data. Along this paper we used the high performance computer DelftBlue^33^ to implement our atlas and fit vine copulas to synthetic data.

## Methods

Since our data relates to graphs, we introduce the basic definitions required for characterizing regular vines and representing them as matrices. We assume that the reader is familiar with concepts of graph theory and repeat the most important concepts required for our purpose for completeness.

### Definitions

In this section we introduce some basic definitions. A more extended treatment may be found for example in^30^. A vine is a set of nested trees. A *tree* is an undirected acyclic graph. More formally, a connected graph *T* = {*N*, *E*} is called a *labeled tree* with nodes *N* = {1, 2…, *d*} and edges *E*, where *E* is a subset of pairs of *N* with no cycle. In this paper the interest is on regular vines.

A *regular vine V* on *d* elements (edge or nodes) is a sequence of trees ${T}_{1},\ldots ,{T}_{d-1}$ such that: (i) *T*_1_ is a tree with node set ${N}_{1}=\{1,\ldots ,d\}$ and edge set *E*_1_, (ii) For $j\ge 2$, *T*_*j*_ is a tree with node set *N*_*j*_ = *E*_*j*−1_ and edge set *E*_*j*_, and (iii) For $j=2,\ldots ,d-1$ and $\{a,b\}\in {E}_{j}$ it must hold that $| a\cap b| =1$. Property (iii) is often referred to as the *proximity condition* which ensures that if there is an edge *e* connecting *a* and *b* in tree *T*_*j*_, $j\ge 2$, then *a* and *b* (which are edges in *T*_*j*−1_) must share a common node in *T*_*j*−1_. Thus, A regular vine on *d* elements is one in which two edges in tree *j* are joined by an edge in tree *j* + 1 only if these edges share a common node in tree *j*.

For *e* ∈ *E*_*j*_, $j\le d-1$, the *constraint set* associated with *e* is the complete union ${U}_{e}^{* }$ of *e*, that is, the subset of ${N}_{1}=\{1,\ldots ,d\}$ reachable from *e* by the membership relation.

**Fig. 1:**
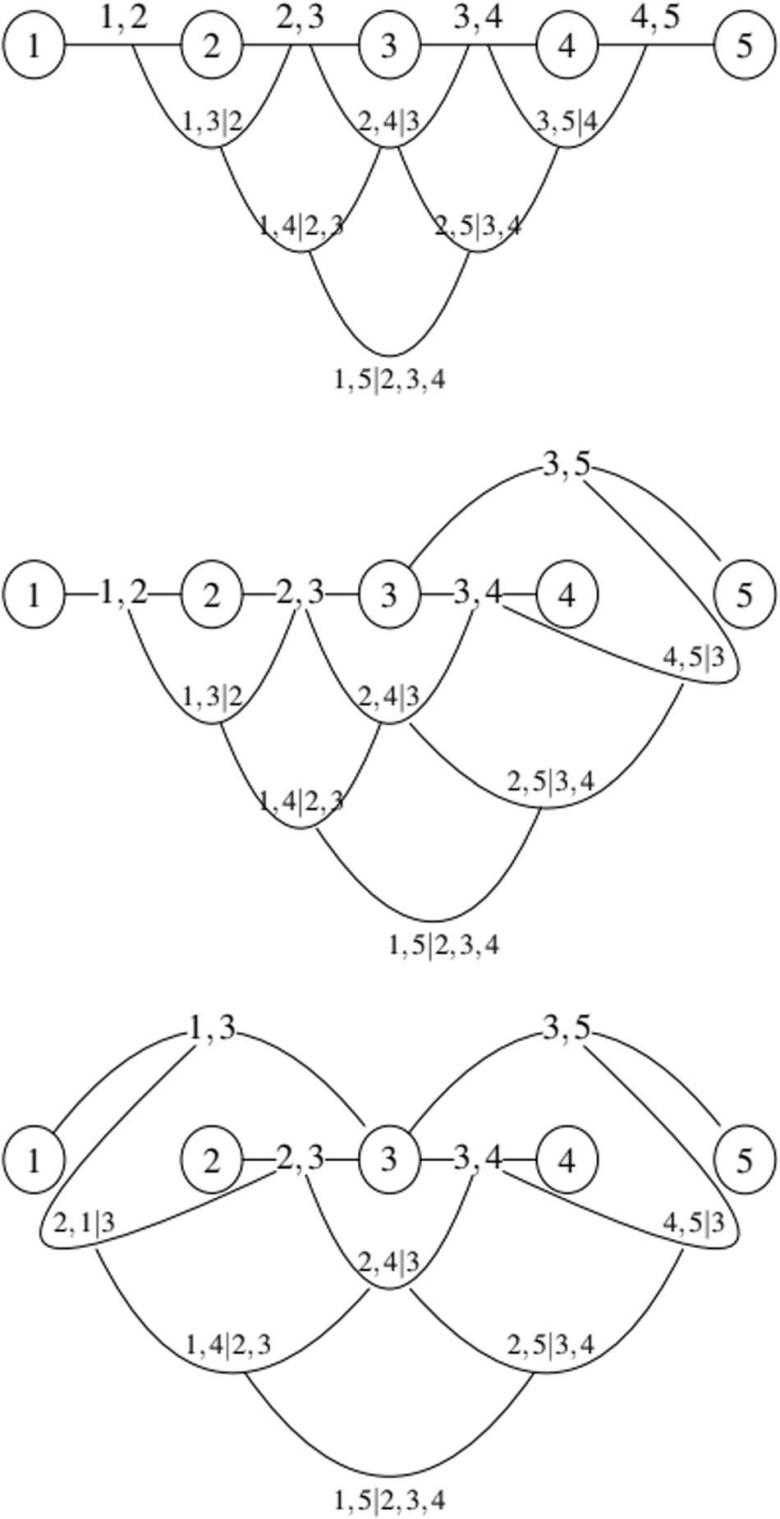
Examples of regular vines on 5 nodes.

For $j=1,\ldots ,d-1$, $e\in E$ if $e=\{i,k\}$ then the *conditioning set* associated with *e* is ${D}_{e}=\left\{{U}_{i}^{* }\cap {U}_{k}^{* }\right\}$ and the *conditioned set* associated with *e* is $\left\{{C}_{e,i},{C}_{e,k}\right\}=\left\{{U}_{i}^{* }\backslash {D}_{e},{U}_{k}^{* }\backslash {D}_{e}\right\}$. Note that for $e\in {E}_{1}$, the conditioning set is empty. Note as well that the order of an edge is the cardinality of its conditioning set. For $e\in {E}_{j}$, $j\le d-1$, $e=\{i,k\}$ we have ${U}_{e}^{* }={U}_{i}\cup {U}_{k}^{* }$. Thus, nodes of *T*_1_ reachable from a given edge via the membership relation are elements of the constraint set of that edge. When two edges in *T*_*j*_ are joined by an edge in *T*_*j*+1_, the intersection of the respective constraint sets forms the conditioning set. The symmetric difference of the constraint sets is the conditioned set of this edge. Figure 1 presents examples of regular vines on 5 elements. Note that the conditioned and conditioning set are presented as ${C}_{e,i},{C}_{e,k}| {D}_{e}$.

Regular vines can be stored as matrices to facilitate their manipulation. The matrix representation was introduced to show that the number of regular vines on *d* nodes is $$\frac{d!}{2}\times {2}^{\left(\begin{array}{c}d-2\\ 2\end{array}\right)}$$^27^. The matrix representation is used in software implementations in R^30^, Python^32^ and matlab^31^. Our data consists precisely of all 24 4 × 4 matrices representing regular vines on 4 nodes, 480 5 × 5 matrices representing regular vines on 5 nodes, 24,030 6 × 6 matrices representing regular vines on 6 nodes, 2,580,480 7 × 7 matrices representing regular vines on 7 nodes and 660,602,880 8 × 8 matrices representing regular vines on 8 nodes.

Since R is by far, the most widely used implementation, we follow the definition provided in^30^ of a regular vine matrix. Let *M* be an upper triangular matrix with entries *m*_*i, j*_ for *i*≤*j*. The elements *m*_*i, j*_ take values in {1, …, *d*}. The matrix *M* is called a *regular vine matrix* or a *matrix representation of a regular vine*, if it satisfies the following conditions:

1. $\left\{{m}_{1,i},\ldots ,{m}_{i,i}\right\}\subset \left\{{m}_{1,j},\ldots ,{m}_{j,j}\right\}$ for $1\le i\le j\le d$. This means that, the entries of a specific column are also contained in all columns right of this column.
2. *m*_*i,i*_ ∋ {*m*_1, *I*_,…, *m*_*i*−1, *i*−1_}. This means that, the diagonal entry of a column does not appear in any column further to the left.
3. For *i* = 3, …, *d* and *k* = 1, …, *i*−1 there exist (*j, l*) with $j\le i$ and $l\le j$ such that $\{{m}_{k,i},\{{m}_{1,i},\ldots ,{m}_{k-1,i}\}\}=\{{m}_{j,j},\{{m}_{1,j},\ldots ,{m}_{l,j}\}\}$ or $\{{m}_{k,i},\{{m}_{1,i},\ldots ,{m}_{k-1,i}\}\}=\{{m}_{l,j},\{{m}_{1,j},\ldots ,{m}_{l-1,j},{m}_{j,j}\}\}$. This last statement means that the elements of *M* should comply with the proximity condition for regular vines.

The regular vine matrices for the examples in Fig. 1 are:

$$A=\left[\begin{array}{ccccc}5 & 5 & 4 & 3 & 2\\  & 4 & 5 & 4 & 3\\  &  & 3 & 5 & 4\\  &  &  & 2 & 5\\  &  &  &  & 1\end{array}\right],\;B=\left[\begin{array}{ccccc}5 & 5 & 3 & 3 & 2\\  & 3 & 5 & 4 & 3\\  &  & 4 & 5 & 4\\  &  &  & 2 & 5\\  &  &  &  & 1\end{array}\right],\;C=\left[\begin{array}{ccccc}5 & 5 & 3 & 3 & 3\\  & 3 & 5 & 4 & 2\\  &  & 4 & 5 & 4\\  &  &  & 2 & 5\\  &  &  &  & 1\end{array}\right].$$

Where matrix A corresponds to the vine in the top of in Fig. 1, matrix B corresponds to the vine in the middle and matrix C corresponds to the vine at the bottom in Fig. 1. For example, the edges of *T*_1_ of the first regular vine in Fig. 1 correspond to $\left\{({a}_{5,5},{a}_{1,5}),({a}_{4,4},{a}_{1,4}),({a}_{3,3},{a}_{1,3}),({a}_{2,2},{a}_{1,2})\right\}=\left\{(1,2),(2,3),(3,4),(4,5)\right\}$. The edges of *T*_2_ for the same figure correspond to $\left\{({a}_{5,5},{a}_{2,5}| {a}_{1,5}),({a}_{4,4},{a}_{2,4}| {a}_{1,4}),({a}_{3,3},{a}_{2,3}| {a}_{1,3})=(1,3| 2),(2,4| 3),(3,5| 4)\right\}$. For *T*_3_, edges are given by $\left\{({a}_{5,5},{a}_{3,5}| {a}_{2,5},{a}_{1,5}),({a}_{4,4},{a}_{3,4}| {a}_{2,4},{a}_{1,4})\right\}=\left\{(1,4| 3,2),(2,5| 4,3)\right\}$. The single edge of *T*_4_ for this regular vine is given by $\left\{({a}_{5,5},{a}_{4,5}| {a}_{3,5},{a}_{2,5},{a}_{1,5})\right\}=\left\{(1,5| 4,3,2)\right\}$. Chimera stores regular vines as matrices, following the definition of *regular vine matrix* presented above and exemplified with the first regular vine in Fig. 1 and its representation as regular vine matrix *A*. More details about how the matrices are presented in Chimera will be shown later in section Data Records.

The first catalogues classifying regular vines are presented in^27^ for up to 7 elements and in^28^ for up to 8 elements. Those catalogues however do not present data corresponding to the regular vine matrices of all vines but only enumerate them. The construction of those catalogues consisted in roughly: i) generate all trees in the first level of the regular vine through Prüfer codes^34^ (see section Technical Validation for a description of Prüfer’s procedure), and ii) construct the line graph (below a definition of line graph) of each tree recursively in the regular vine and find all possible spanning trees of each tree of the regular vine. This procedure warranties the uniqueness of each vine. The procedure followed to construct Chimera is similar to the one presented in^27^ and^28^ except it does not use Prüfer codes. It however still relies on the concept of a line graph.

Given a graph *G* = (*N, E*), its *line graph L*(*G*) is a graph $({N}_{\ell },{E}_{\ell })$ such that:

- Every $e\in E$ corresponds to an ${n}_{\ell }\in {N}_{\ell }$ and,
- ${n}_{i},{n}_{j}\in {N}_{\ell }$, with $i\ne j$ are adjacent if and only if their corresponding edges share a common endpoint (“are incident”) in *G*.

**Fig. 2:**
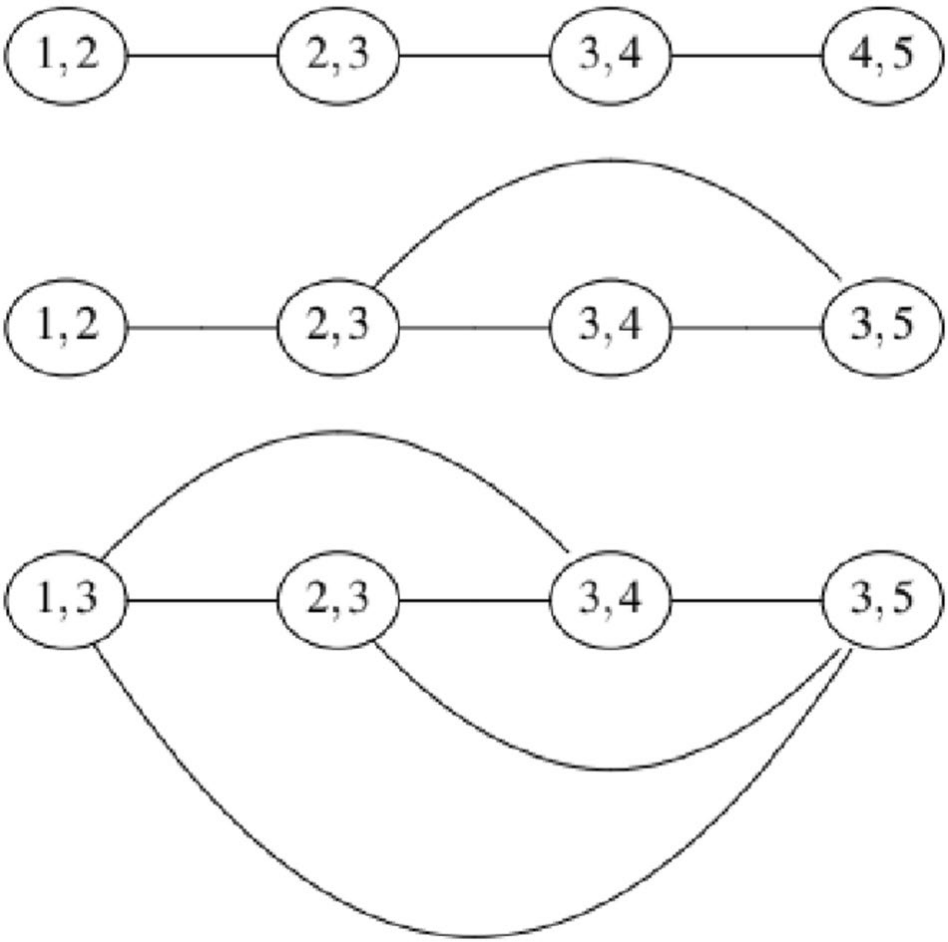
Line graphs for the first tree of the regular vines in Fig. 1.

That is, *L*(*G*) is the intersection graph of the edges of *G*, representing each edge by the set of its two endpoints. Notice that by definition, all spanning trees of the line graph will comply with the regularity condition for vines. Line graphs are also known as derived graphs, interchange graphs, adjoin and edge to vertex dual. Harary^35^ notes that the concept of the line graph of a given graph is so natural that it has been rediscovered independently by many authors. The line graphs of the first tree of the regular vines presented in Fig. 1 are shown in Fig. 2. Notice that the first line graph shown in Fig. 2 has only one spanning tree. These type of graphs are usually referred to as “lines” while the line graph of the first tree of the third regular vine shown in Fig. 1 (which is usually referred to as a “star”) is a *complete graph* (all nodes are adjacent to each other) and hence it has 4^4−2^ = 16 spanning trees.

The steps taken to generate all regular vine matrices contained in Chimera are:

1. A library of *non-isomorphic* trees is constructed. Two graphs *G* = {*V*, *E*} and *H* = {*W*, *F*} are isomorphic if there is bijective function *f* : *V*→*W* such that ∀ ${v}_{1},{v}_{2}\in V$, $\{{v}_{1},{v}_{2}\}\in E\iff \{f({v}_{1}),f({v}_{2})\}\in F$. Loosely speaking, two trees are non-isomorphic if they do not have the same structure. This library constructed for Chimera consists of the 45 trees presented in Table S1 of the supplement. The trees are denoted T4, T5, …, T47, T48. Notice that by labeling these trees through different permutations all possible trees on 4 up to 8 nodes are obtained.
2. Starting with a complete graph on *d* nodes (see the definition of a complete graph above), all *d*^*d*−2^ labelled trees on *d* nodes are found by brute force. Arthur Cayley^36^ was the first to note that for every positive integer *d*, the number of trees on *d labeled* nodes is *d*^d−2^. For any labeled complete graph with *d* nodes, the number of spanning trees of this graph must be thus *d*^*d*−2^. For example, the line graph at the bottom of Fig. 2 is a complete graph (all nodes share an edge with each other) on 4 nodes. This graph must have 16 labeled spanning trees of which 4 are of the type T5 in Table S1 of the supplement and 12 are of the type T4 in the same table. Once all trees for the first level of the regular vine are found, they are categorized according to their non-isomorphic tree from step 1. For example, T4 in Table S1 of the supplement will have $\frac{4!}{2}=12$ ways of being labelled. That is, all possible permutations of numbers in 1, 2, 3, 4 divided by 2 to avoid repetitions (for example, a tree 1-2-3-4 is equal to 4-3-2-1 hence this permutation must not be double counted). Similarly T5 in Table S1 of the supplement, has 4 possible ways to be labeled assigning the number 1, …, 4 to the node adjacent to all other nodes. These will be used as the trees in the first level of the regular vines.
3. At this step Prüfer codes are also obtained for each labeled tree. See section technical validation below where Prüfer codes are discussed. Steps 1 to 3 are performed using the Python script geninput.py which is available in the 4TU data repository under the Python data collection^37^.
4. For each non-isomorphic tree in step 1, a line graph is constructed for the edges of the tree in the first level of the regular vine, and all spanning trees of this graph are obtained again by brute force. For example, the line graph of *T*_1_ in the first regular vine of Fig. 1 is the first graph presented in Fig. 2. Notice that this line graph is a tree (a so called line) and has only one spanning tree (which is the graph itself). The line graph of *T*_1_ of the second regular vine is the second graph in Fig. 2. This graph has 3 spanning trees. The edge sets of these spanning trees are {{(1, 2), (2, 3)}, {(2, 3), (3, 4)}, {(3, 4), (3, 5)}}, {{(1, 2), (2, 3)}, {(2, 3), (3, 5)}, {(3, 5), (3, 4)}} and {{(1, 2), (2, 3)}, {(2, 3), (3, 5)}, {(2, 3), (3, 4)}}. *T*_1_ of the third regular vine shown in Fig. 1 is a so called star (all edges share a common node which is node 3 in this case). Its line graph is the complete graph shown at the bottom of Fig. 2 which as explained in step 2 above has 4^2^ = 16 spanning trees.
5. Step 4 is repeated for each tree in each level of the regular vine until the last level of the vine. The results are written as a regular vine matrix if the first tree of the vine corresponds to a line (such as in the first regular vine presented in Fig. 1) or matrices whenever the first tree of the regular vine is not a line. Notice that at this point regular vines are classified according to their tree-equivalent class. Two vines are *tree-equivalent* if they share the same non-isomorphic tree in each level of the vine. For example by permuting nodes 4 and 5 in the first regular vine shown in Fig. 1, two distinct regular vines (and hence regular vine matrices) are obtained. However, these fall in the same tree-equivalent class. Notice that by permuting nodes 4 and 5 in the second and third regular vines shown in Fig. 1 exactly the same regular vines (and hence regular vine matrices) are obtained. However by permuting nodes 5 and 3 (for example), distinct regular vines within the same tree equivalent class will be obtained respectively. Tree-equivalent classes for all regular vines on up to 8 nodes are presented through their tree sequence in Table S2 of the supplement. The number of distinct regular vines (and regular vine matrices) within each tree equivalent class is also shown in the same table.
6. Finally all regular vines (and consequently their matrix representation) within each tree equivalent class are found through permutation. Steps 4 to 6 are performed using the Python script genmatrix.py which is available in the 4TU data repository under the Python data collection^37^. This script was specifically modified and implemented for use in the high performance computer DelftBlue^33^ of the Technical University of Delft.

### Using all regular vine matrices in Chimera to fit vine copulas to synthetic data

Vine copulas characterize complex multidimensional probability distributions. In real-case applications, the structure of the vine copula (e.g., trees and bi-variate dependence) is fitted (and its goodness of fit evaluated) based on available observations. In our case, to illustration the possibilities of Chimera, we fit all vine copulas in 4, 5, 6, 7 and 8 variables to synthetic data. Five synthetic data sets, of 1000 observations each, are generated with regular vines. The details are given in section 2 of the supplement. For example, in section 2.1.1 of the supplement, 1000 samples are generated from a regular vine whose first tree is 2-3-1-4 (see *M*_1_) with bi-variate copulas and parameters shown in Tables S3, S4. All 24 vine copulas on 4 variables are fitted to the synthetic data using the 24 regular vine matrices representing regular vines on 4 nodes included in Chimera. The selected fit through a brute-force procedure, that is, the one with minimum Akaike’s Information Criterion (AIC), is also shown as *R*_1_ in section 2.1.2 of the supplement. Tables S6, S7 of the supplement show the bi-variate copulas and parameters corresponding to *R*_1_. Notice that in this case a brute-force procedure is able to find the regular vine which is used originally to generate the synthetic data. The Python package “pyvinecopulib”^32^ was used.

**Fig. 3:**
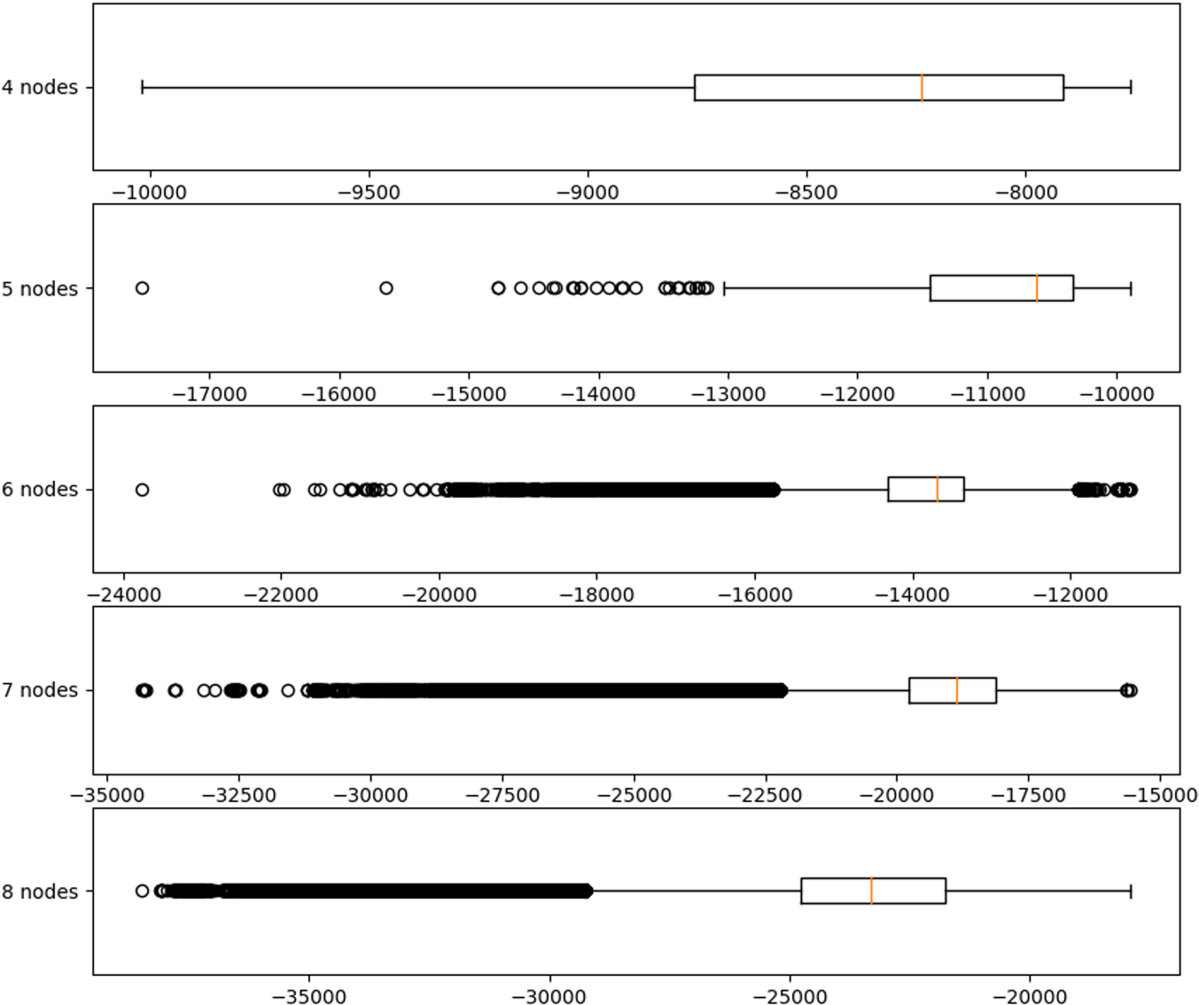
Box plots of Akaike’s Information Criteria for fits of all regular vines included in Chimera.

This process was repeated for synthetic datasets with 5, 6, 7 and 8 variables. Notice that in most cases a brute-force procedure based on AIC is able to capture the regular vine that generates the synthetic data except for 7 variables where *M*_4_ ≠ *R*_4_. The copulas in each tree are not always captured exactly. However, general characteristics (upper or lower tail dependence for example) of the joint distribution are. Datasets on 4 and 5 variables can be fitted relatively easily (depending on the sample size) in a personal computer with the aid of Chimera. Relatively small samples (300 for example) of a 6 dimensional distribution can be fitted within days in a standard personal computer. In order to fit 7 and 8 dimensional vine copulas to data the DelftBlue supercomputer was used. Notice that the computational time required to fit all vine copula models on 8 elements to the sample, amounts to approximately 12 years (Table S29 in section 3.4 of the supplement). Fitting all vine copula models to 1000 samples of a 7-dimensional data set in the DelftBlue super computer is a matter of hours when computing on parallel. The fitting of vine copulas on 8 variables is however more challenging and takes days of parallel computing rather than hours. A more extended discussion of the computational challenges of fitting vine copulas on 7 or more variables is presented in section 3 of the supplement. A box plot showing AIC for all vine copula models that use regular vines (represented by their regular vine matrices) included in Chimera to synthetic data is presented in Fig. 3. An investigation of one of the most commonly used fitting algorithms^26^ for vine copulas on up to 8 nodes using Chimera is the subject of recent research by the authors.

## Data Records

Our atlas Chimera is hosted in the 4TU research data repository^37^. For the different platforms (R, matlab and Python) different files are available. The data containing regular vine matrices was originally created in Python and then transformed to R and matlab formats. The naming convention for the available files is presented in Table 1.

**Table 1:** Naming convention for files containing regular vine matrices in Chimera.

Platform	Naming convention
Python	submat_<Number of nodes>_<type of tree>.pbz2
matlab	submat_<Number of nodes>_<type of tree>Matlab.mat
R	submat_<Number of nodes>_<type of tree>R.RData

**Fig. 4:**
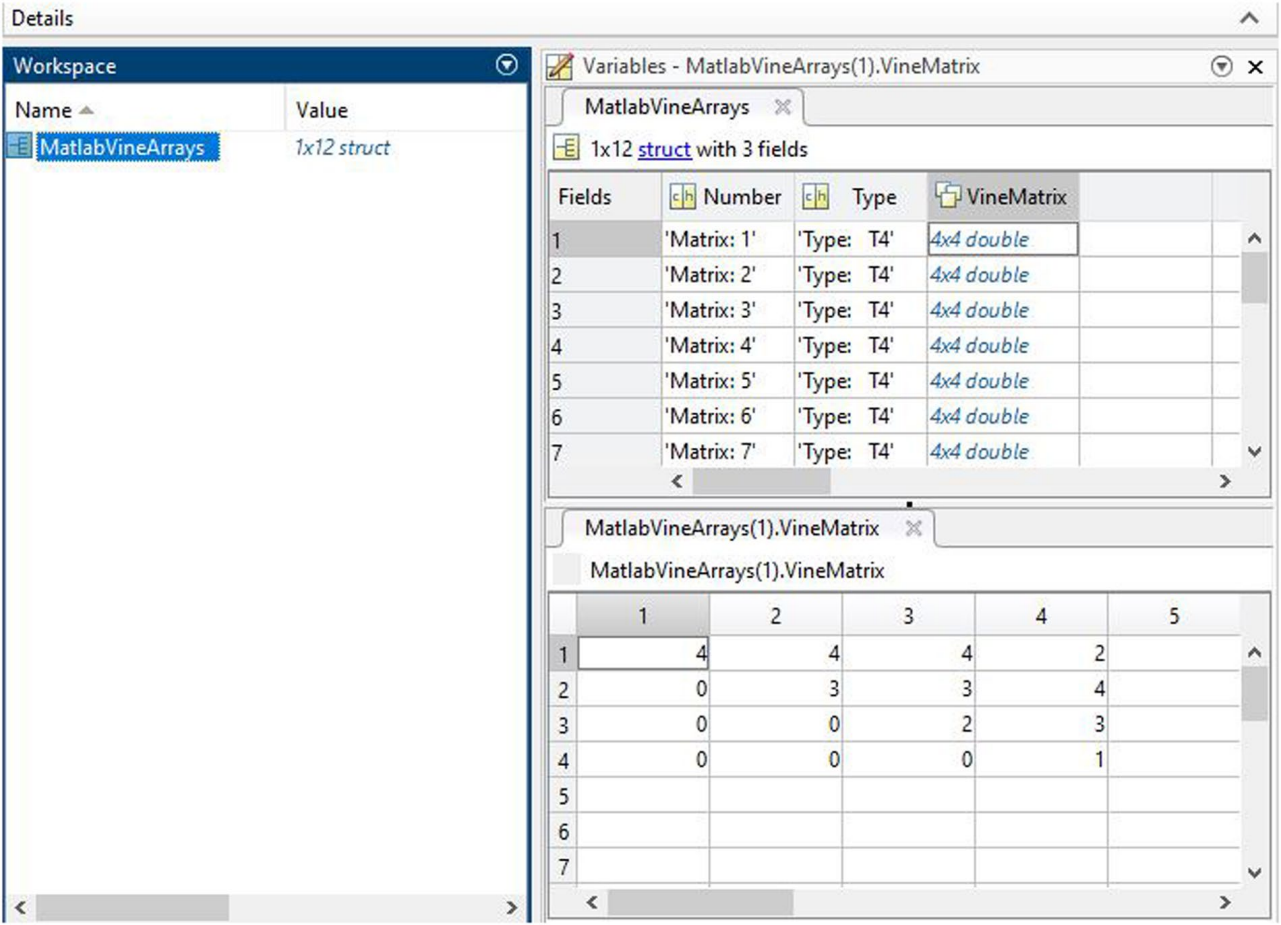
matlab dataset capture.

Figure 4 shows a screen shot of file submats_4_T4Matlab.mat. The matlab data in Fig. 4 is a structure array named “MatlabVineArrays”. It contains a total of 12 elements, each with 3 fields. The “Type”, which corresponds to a tree-equivalent regular vine class, the regular vine matrix number (“Number”) and the matrix (named “VineMatrix”) itself. The tree-equivalent class refers to the tree sequence corresponding to the particular tree in each level of the regular vine. The non-isomorphic trees used in the construction of tree-equivalent regular vines included in Chimera are presented in the supplement.

Table S1 of the supplement presents non-isomorphic trees (and their labels) used in the construction of each regular vine included in Chimera. Table S2 of the supplement presents: (i) all tree-equivalent classes (using the tree sequence), (ii) the naming convention (with Python extension) and (iii) the number of regular vine matrices included in each tree-equivalent class. There are a total of 22 matlab files submat_4_T4Matlab.mat,…, submats_7_T25Matlab.mat which contain all regular vine matrices for regular vines on 4, …, 7 nodes. All together the 22 matlab files occupy ≈40 Mb.

**Fig. 5:**
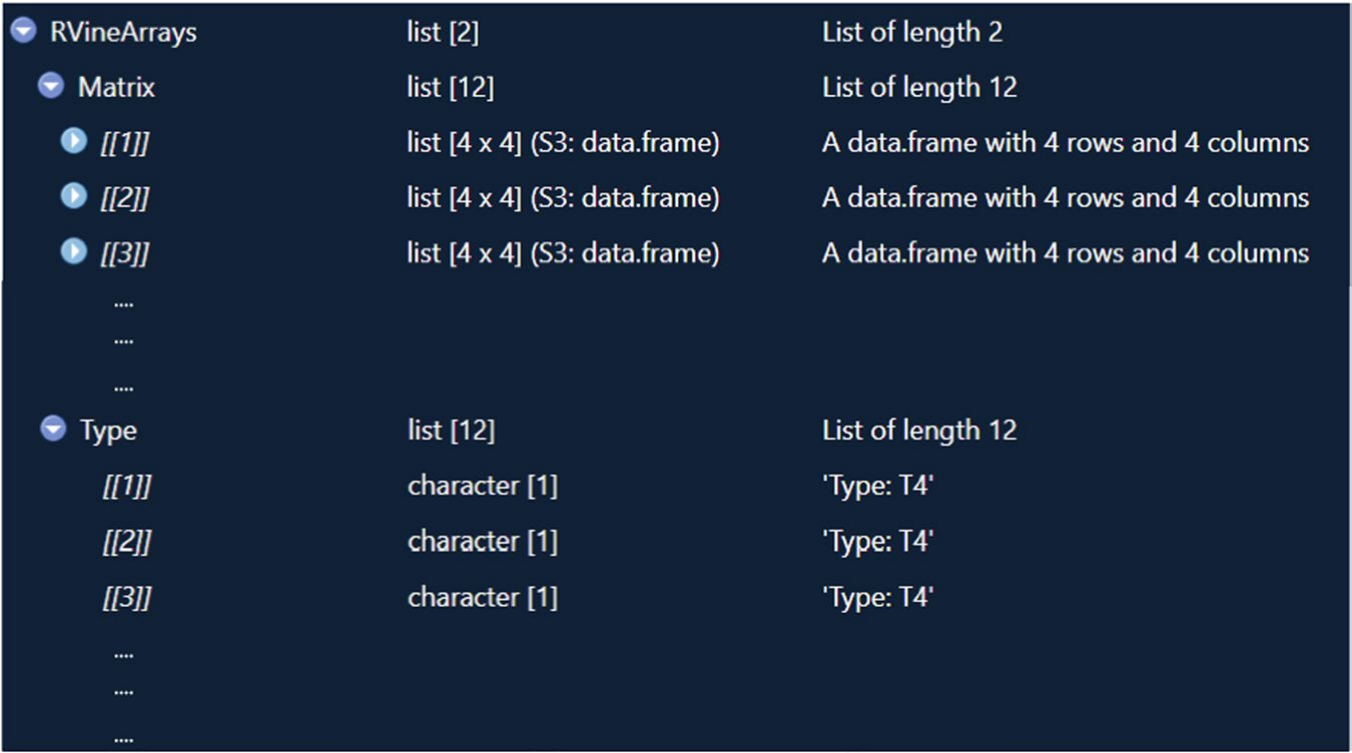
R dataset capture.

Figure 5 shows a representation of the dataset in R. The data is ordered within lists, the main list is called “RVineArrays” and the nested lists contain the vine matrices (“Matrix”) and their respective tree sequences (“Type”).

For Python, the extension of the file is “pbz2”, because the amount of data increases drastically after 7 nodes (the total size of the Python data is ≈3.9 Gb). The initial ascii files are compressed using the cPickle module in Python and supplied in a digital format. An example Python script is included to retrieve data from binary files (see section Code Availability). Essentially, each matrix available in the file is presented with an index number (“index”), the tree type in the first level of the vine (“mat_type”) and the matrix (“matrix”) to be used within pyvinecopulib^32^ which is the Python library available for operating with regular vines.

Finally, all files available for Python are presented in Table S2 in the supplement. The 660,602,880 8 × 8 matrices representing regular vines on 8 nodes are only available for Python. To construct the regular vine matrices with the methods described above, the high performance cluster (supercomputer) DelftBluePhase1^33^ of the Technical University of Delft was used using parallel processing.

For example, as may be seen in Table S2 in the supplement, for the Python data set, submat_7_T25.pbz2 will contain all regular vine matrices whose first tree corresponds to T25 (shown in Table S1 of the supplement). A total of 22 distinct tree-equivalent regular vines (tree sequences) have T25 in the first tree of their tree-sequence. There are a total of 161,280 regular vine matrices distributed among the 22 tree-equivalent classes.

There are 576 times more regular vines on 9 nodes than there are on 8. There are also 737,280 times more regular vines on 10 nodes than there are on 8. It is not clear at the moment to the authors the computational, processing and storage restrictions required to extend Chimera to include regular vine matrices on 9 and 10 elements. It is also unclear at the moment to the authors the feasibility of using an extended catalogue in practice. These will be however subject of future research by the authors and hopefully by other research groups interested in Chimera.

## Technical Validation

Notice that the application of the methods described in section Methods warranty the construction of all unique regular vine matrices. The procedure described in section Methods generates labelled trees through brute force. By obtaining Prüfer codes in step 3 of the procedure to generate regular vines we make sure that we have taken into account exactly *d*^*d*−2^ labelled trees to construct the regular vines in Chimera.

Prüfer’s procedure is based on the fact that there is a one to one correspondence between the set of trees with *d* labeled nodes and sequences of integers in {1, …, *d*} of length *d*−2. In his paper Prüfer obtains the correspondence by the following procedure: for a given tree, remove the endpoint with the smallest label (other than the root). The endpoints are nodes with degree one in the tree, they are sometimes referred to as *leafs*. Choose for example *d* as the root. Choosing any other node as the root would not change the procedure except the labelling of trees. Then, let ${\ell }_{1}$ be the label of the unique node which is adjacent to it. Remove the endpoint and the edge adjacent to it to obtain a tree on *d*−1 nodes. Repeat the operation with the new tree on *d*−1 nodes to obtain ${\ell }_{2}$ and so on. The process is terminated when a tree on two nodes has been found. The reader may check that the trees on the first level of the regular vines shown in Fig. 1 have Prüfer codes (2, 3, 4), (2, 3, 3) and (3, 3, 3) respectively.

The catalogues presented in^27^ and^28^ enumerate regular vines though Prüfer codes rather than the brute force procedures described in the Methods section. Notice that the number of regular vine matrices available in Chimera presented per tree-equivalence class in Table S2 of the supplement, coincide exactly with the enumeration presented in^27^ and^28^ that was obtained through different procedures. Finally as observed in section Using all regular vine matrices in Chimera to fit vine copulas to synthetic data, all regular vine matrices included in Chimera were used to fit vine copulas to synthetic data using the Python package “pyvinecopulib”^32^ resulting in unique goodness of fit measures based on likelihood such as Akaike’s Information Criterion (AIC).

## Supplementary information

Supplement to "Chimera: an atlas of regular vines on up to 8 nodes"

## Data Availability

The scrips used to generate regular vine matrices in Python are included in the 4TU data repository under the Python data collection^37^ (see the Methods section). The files containing regular vine matrices on up to 8 nodes for Python are compressed files in pbz2 format. In order to use these files, these need to be decompressed. For future users of the dataset a specific script get_matrices.py is available together with the files in the repository^37^. This script provides an example, contains subroutines and the Python tree-equivalent class definition for each one of the matrices of interest. Roughly, what the get_matrices.py script will do is get the matrices from files in a user specified directory for the specified number of nodes. By default, an array is returned with all matrices as a Python class, containing the tree-equivalent class (tree sequence type), index and matrix. For convenience, a user can also specify parts of the dataset based on the tree-equivalent class, which relates to the files names of the dataset.
