# Supplementary material for: Chimera: An atlas of regular vines on up to 8 nodes
Source: Sci Data. 2023 May 31;10:337. doi: 10.1038/s41597-023-02252-6 (PMC10230490; doi:10.1038/s41597-023-02252-6)
Supplement: Supplementary file 1 — Supplement to "Chimera: an atlas of regular vines on up to 8 nodes" [file 41597_2023_2252_MOESM1_ESM.pdf]

Supplement to "Chimera: an atlas of regular vines on up to 8 nodes"

Oswaldo Morales-Nápoles[1,**], Mojtaba Rajabi-Bahaabadi[2], Gina Alexandra Torres-Alves[1], and Marcel 't Hart[1,3]

[1]Delft University of Technology, Hydraulic Engineering, Delft, 2645GX, The Netherlands
[2]Yazd University, Civil Engineering, Yazd, 8915818411, Iran
[3]Tunnel Engineering Consultants, Amersfoort, The Netherlands
[**]corresponding author: Oswaldo Morales-Nápoles (o.moralesnapoles@tudelft.nl)

May 17, 2023

Pasemos, ahora, del jardín zoológico de la realidad al jardín zoológico de las mitologías, al jardín cuya fauna no es de leones sino de esfinges y de grifos y de centauros. La población de este segundo jardín debería exceder a la del primero, ya que un monstruo no es otra cosa que una combinación de elementos de seres reales y que las posibilidades del arte combinatorio lindan con lo infinito.
Manual de zoología fantástica. J.L.Borges

Contents

1 File names, tree sequences and number of regular vine matrices included in Chimera 3

2 Synthetic data and regular vines with smallest AIC after fitting all regular vines included in Chimera 43

2.1 Regular vines on 4 nodes 43

2.1.1 Regular vine used to generate synthetic data 43

2.1.2 Regular vine on 4 elements with smallest AIC after fitting all regular vines on 4 nodes. 43

2.2 Regular vines on 5 nodes 45

2.2.1 Regular vine used to generate synthetic 45
2.2.2 Regular vine on 5 elements with smallest AIC after fitting all regular vines on 5 nodes. 45
2.3 Regular vines on 6 nodes 47
2.3.1 Regular vine used to generate synthetic 47
2.3.2 Regular vine on 6 elements with smallest AIC after fitting all regular vines on 6 nodes. 47
2.4 Regular vines on 7 nodes 49
2.4.1 Regular vine used to generate synthetic 49
2.4.2 Regular vine on 7 elements with smallest AIC after fitting all regular vines on 7 nodes. 50
2.5 Regular vines on 8 nodes 51
2.5.1 Regular vine used to generate synthetic 51
2.5.2 Regular vine on 8 elements with smallest AIC after fitting all regular vines on 8 nodes. 52

3 Brute-force computational process to find the best fit for 4, 5, 6, 7 and 8 nodes using Chimera 53
3.1 Introduction 53
3.2 Parallel processing 53
3.3 General approach 53
3.4 Calculations 54

List of Tables

S1 Non-isomorphic trees on 4, 5, 6, 7 and 8 nodes and their labels . 4
S2 File name, tree sequence and number of matrices per tree sequence for regular vines on 4 to 8 nodes 5
S3 Bivariate copulas corresponding to the trees on each level of the regular vine corresponding to M_1 43
S4 Parameters for the bivariate copulas in Table S3 43
S5 General characteristics of brute-force fitting regular vines on 4 nodes 43
S6 Bivariate copulas corresponding to the trees on each level of the regular vine corresponding to R_1 44
S7 Parameters for the bivariate copulas in Table S6 44
S8 Bivariate copulas corresponding to the trees on each level of the regular vine represented by M_2 45
S9 Parameters for the bivariate copulas in Table S8 45
S10 General characteristics of brute-force fitting regular vines on 5 nodes 45
S11 Bivariate copulas corresponding to the trees on each level of the regular vine corresponding to R_2 46
S12 Parameters for the bivariate copulas in Table S11 46

S13 Bivariate copulas corresponding to the trees on each level of the regular vine corresponding to M_3 47
S14 Parameters for the bivariate copulas in Table S13 47
S15 General characteristics of brute-force fitting regular vines on 5 nodes 47
S16 Bivariate copulas corresponding to the trees on each level of the regular vine corresponding to R_3 48
S17 Parameters for the bivariate copulas in Table S16 48
S18 Bivariate copulas corresponding to the trees on each level of the regular vine corresponding to M_4 49
S19 Parameters for the bivariate copulas in Table S18 49
S20 General characteristics of brute-force fitting regular vines on 7 nodes 50
S21 Bivariate copulas corresponding to the trees on each level of the regular vine corresponding to R_4 50
S22 Parameters for the bivariate copulas in Table S21 50
S23 Bivariate copulas corresponding to the trees on each level of the regular vine corresponding to M_5 51
S24 Parameters for the bivariate copulas in Table S23 51
S25 General characteristics of brute-force fitting regular vines on 8 nodes 52
S26 Bivariate copulas corresponding to the trees on each level of the regular vine corresponding to R_5 52
S27 Parameters for the bivariate copulas in Table S26 52
S28 Number of files used to fit all regular vines included in Chimera to synthetic data 54
S29 Calculation times for fitting all regular vines included in Chimera to synthetic data 55

1 File names, tree sequences and number of regular vine matrices included in Chimera

In this supplement we present the non-isomorphic trees used in the construction of Chimera. Table S1 presents the 44 non-isomorphic trees on 4, 5, 6, 7 and 8 nodes with their respective labels. Tree-equivalent regular vines are presented in Table S2 according to their tree sequence. Each tree from Table S1 will be displayed in order after the + sign for the construction of vines. There is one tree-equivalent regular vine on 3 nodes. Every regular vine on d nodes for $d > 3$ uses the regular vine on three nodes in its construction. For this reason T3 + T2 + T1 (which are the trees on 3, 2 and 1 nodes respectively) will be omitted when indicating the sequence of trees used in the construction of different tree-equivalent regular vines. For example the D-vine on 4 nodes will be V4 = T4 + T3 + T2 + T1 = T4. Tables S1 and S2 are based on the catalogues presented

in [5, 6]. Table S2 differs from the catalogues in [5, 6] in that it additionally presents the naming convention for the files contained in Chimera.

Table S1: Non-isomorphic trees on 4, 5, 6, 7 and 8 nodes and their labels

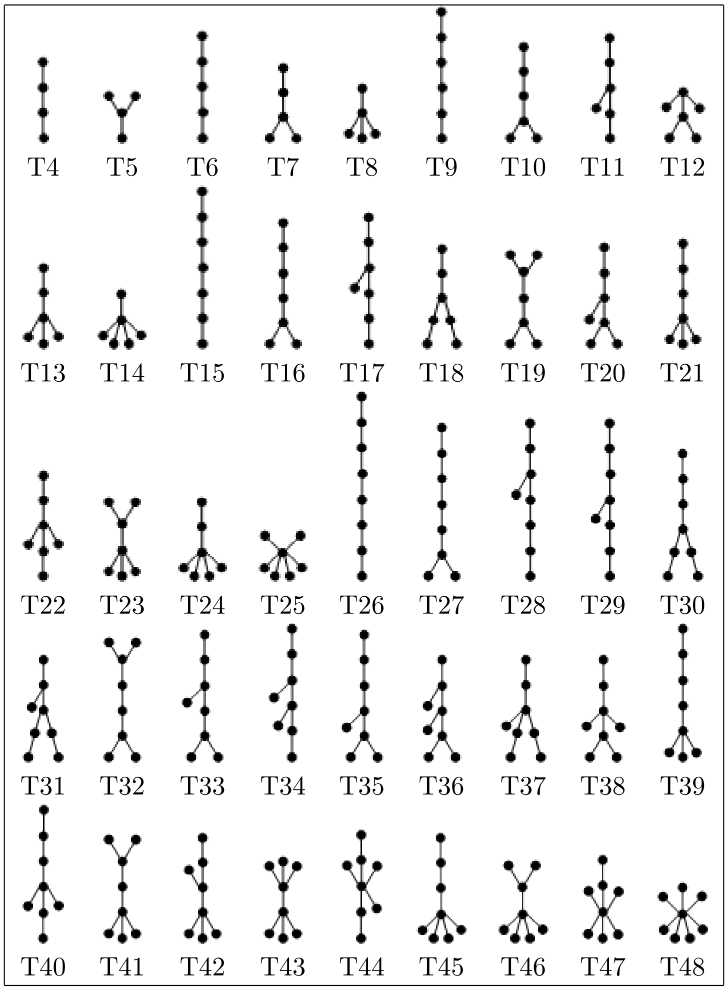

Table S2: File name, tree sequence and number of matrices per tree sequence for regular vines on 4 to 8 nodes

File name	Tree Sequence	# matrices
submats_4_T4.pbz2	T4	12
submats_4_T5.pbz2	T5	12
submats_5_T6.pbz2	T6+T4	60
submats_5_T7.pbz2	T7+T4	120
submats_5_T7.pbz2	T7+T5	180
submats_5_T8.pbz2	T8+T4	60
submats_5_T8.pbz2	T8+T5	60
submats_6_T10.pbz2	T10+T6+T4	720
submats_6_T10.pbz2	T10+T7+T4	720
submats_6_T10.pbz2	T10+T7+T5	1080
submats_6_T11.pbz2	T11+T6+T4	360
submats_6_T11.pbz2	T11+T7+T4	1440
submats_6_T11.pbz2	T11+T7+T5	2160
submats_6_T12.pbz2	T12+T6+T4	360
submats_6_T12.pbz2	T12+T7+T4	720
submats_6_T12.pbz2	T12+T7+T5	1080
submats_6_T12.pbz2	T12+T8+T4	1080
submats_6_T12.pbz2	T12+T8+T5	1080
submats_6_T13.pbz2	T13+T6+T4	720
submats_6_T13.pbz2	T13+T7+T4	2160
submats_6_T13.pbz2	T13+T7+T5	3240
submats_6_T13.pbz2	T13+T8+T4	1440
submats_6_T13.pbz2	T13+T8+T5	1440
submats_6_T14.pbz2	T14+T6+T4	360
submats_6_T14.pbz2	T14+T7+T4	720
submats_6_T14.pbz2	T14+T7+T5	1080
submats_6_T14.pbz2	T14+T8+T4	360
submats_6_T14.pbz2	T14+T8+T5	360
submats_6_T9.pbz2	T9+T6+T4	360
submats_7_T15.pbz2	T15+T9+T6+T4	2520
submats_7_T16.pbz2	T16+T10+T6+T4	5040
submats_7_T16.pbz2	T16+T10+T7+T4	5040
submats_7_T16.pbz2	T16+T10+T7+T5	7560
submats_7_T16.pbz2	T16+T9+T6+T4	5040
submats_7_T17.pbz2	T17+T10+T6+T4	10080
submats_7_T17.pbz2	T17+T10+T7+T4	10080
submats_7_T17.pbz2	T17+T10+T7+T5	15120
submats_7_T17.pbz2	T17+T11+T6+T4	5040
submats_7_T17.pbz2	T17+T11+T7+T4	20160
submats_7_T17.pbz2	T17+T11+T7+T5	30240
submats_7_T17.pbz2	T17+T9+T6+T4	5040

Continued on next page

Table S2 – continued from previous page

File name	Tree sequence	# matrices
submats_7_T18.pbz2	T18+T11+T6+T4	2520
submats_7_T18.pbz2	T18+T11+T7+T4	10080
submats_7_T18.pbz2	T18+T11+T7+T5	15120
submats_7_T19.pbz2	T19+T10+T6+T4	5040
submats_7_T19.pbz2	T19+T10+T7+T4	5040
submats_7_T19.pbz2	T19+T10+T7+T5	7560
submats_7_T19.pbz2	T19+T12+T6+T4	2520
submats_7_T19.pbz2	T19+T12+T7+T4	5040
submats_7_T19.pbz2	T19+T12+T7+T5	7560
submats_7_T19.pbz2	T19+T12+T8+T4	7560
submats_7_T19.pbz2	T19+T12+T8+T5	7560
submats_7_T19.pbz2	T19+T9+T6+T4	2520
submats_7_T20.pbz2	T20+T10+T6+T4	15120
submats_7_T20.pbz2	T20+T10+T7+T4	15120
submats_7_T20.pbz2	T20+T10+T7+T5	22680
submats_7_T20.pbz2	T20+T11+T6+T4	5040
submats_7_T20.pbz2	T20+T11+T7+T4	20160
submats_7_T20.pbz2	T20+T11+T7+T5	30240
submats_7_T20.pbz2	T20+T12+T6+T4	10080
submats_7_T20.pbz2	T20+T12+T7+T4	20160
submats_7_T20.pbz2	T20+T12+T7+T5	30240
submats_7_T20.pbz2	T20+T12+T8+T4	30240
submats_7_T20.pbz2	T20+T12+T8+T5	30240
submats_7_T20.pbz2	T20+T13+T6+T4	15120
submats_7_T20.pbz2	T20+T13+T7+T4	45360
submats_7_T20.pbz2	T20+T13+T7+T5	68040
submats_7_T20.pbz2	T20+T13+T8+T4	30240
submats_7_T20.pbz2	T20+T13+T8+T5	30240
submats_7_T20.pbz2	T20+T9+T6+T4	5040
submats_7_T21.pbz2	T21+T10+T6+T4	5040
submats_7_T21.pbz2	T21+T10+T7+T4	5040
submats_7_T21.pbz2	T21+T10+T7+T5	7560
submats_7_T21.pbz2	T21+T11+T6+T4	5040
submats_7_T21.pbz2	T21+T11+T7+T4	20160
submats_7_T21.pbz2	T21+T11+T7+T5	30240
submats_7_T21.pbz2	T21+T13+T6+T4	5040
submats_7_T21.pbz2	T21+T13+T7+T4	15120
submats_7_T21.pbz2	T21+T13+T7+T5	22680
submats_7_T21.pbz2	T21+T13+T8+T4	10080
submats_7_T21.pbz2	T21+T13+T8+T5	10080
submats_7_T21.pbz2	T21+T9+T6+T4	5040
submats_7_T22.pbz2	T22+T10+T6+T4	10080
submats_7_T22.pbz2	T22+T10+T7+T4	10080

Continued on next page

Table S2 – continued from previous page

File name	Tree sequence	# matrices
submats_7_T22.pbz2	T22+T10+T7+T5	15120
submats_7_T22.pbz2	T22+T11+T6+T4	7560
submats_7_T22.pbz2	T22+T11+T7+T4	30240
submats_7_T22.pbz2	T22+T11+T7+T5	45360
submats_7_T22.pbz2	T22+T12+T6+T4	10080
submats_7_T22.pbz2	T22+T12+T7+T4	20160
submats_7_T22.pbz2	T22+T12+T7+T5	30240
submats_7_T22.pbz2	T22+T12+T8+T4	30240
submats_7_T22.pbz2	T22+T12+T8+T5	30240
submats_7_T22.pbz2	T22+T13+T6+T4	15120
submats_7_T22.pbz2	T22+T13+T7+T4	45360
submats_7_T22.pbz2	T22+T13+T7+T5	68040
submats_7_T22.pbz2	T22+T13+T8+T4	30240
submats_7_T22.pbz2	T22+T13+T8+T5	30240
submats_7_T22.pbz2	T22+T9+T6+T4	2520
submats_7_T23.pbz2	T23+T10+T6+T4	10080
submats_7_T23.pbz2	T23+T10+T7+T4	10080
submats_7_T23.pbz2	T23+T10+T7+T5	15120
submats_7_T23.pbz2	T23+T11+T6+T4	5040
submats_7_T23.pbz2	T23+T11+T7+T4	20160
submats_7_T23.pbz2	T23+T11+T7+T5	30240
submats_7_T23.pbz2	T23+T12+T6+T4	5040
submats_7_T23.pbz2	T23+T12+T7+T4	10080
submats_7_T23.pbz2	T23+T12+T7+T5	15120
submats_7_T23.pbz2	T23+T12+T8+T4	15120
submats_7_T23.pbz2	T23+T12+T8+T5	15120
submats_7_T23.pbz2	T23+T13+T6+T4	20160
submats_7_T23.pbz2	T23+T13+T7+T4	60480
submats_7_T23.pbz2	T23+T13+T7+T5	90720
submats_7_T23.pbz2	T23+T13+T8+T4	40320
submats_7_T23.pbz2	T23+T13+T8+T5	40320
submats_7_T23.pbz2	T23+T14+T6+T4	25200
submats_7_T23.pbz2	T23+T14+T7+T4	50400
submats_7_T23.pbz2	T23+T14+T7+T5	75600
submats_7_T23.pbz2	T23+T14+T8+T4	25200
submats_7_T23.pbz2	T23+T14+T8+T5	25200
submats_7_T23.pbz2	T23+T9+T6+T4	5040
submats_7_T24.pbz2	T24+T10+T6+T4	15120
submats_7_T24.pbz2	T24+T10+T7+T4	15120
submats_7_T24.pbz2	T24+T10+T7+T5	22680
submats_7_T24.pbz2	T24+T11+T6+T4	7560
submats_7_T24.pbz2	T24+T11+T7+T4	30240
submats_7_T24.pbz2	T24+T11+T7+T5	45360

Continued on next page

Table S2 – continued from previous page

File name	Tree sequence	# matrices
submats_7_T24.pbz2	T24+T12+T6+T4	10080
submats_7_T24.pbz2	T24+T12+T7+T4	20160
submats_7_T24.pbz2	T24+T12+T7+T5	30240
submats_7_T24.pbz2	T24+T12+T8+T4	30240
submats_7_T24.pbz2	T24+T12+T8+T5	30240
submats_7_T24.pbz2	T24+T13+T6+T4	20160
submats_7_T24.pbz2	T24+T13+T7+T4	60480
submats_7_T24.pbz2	T24+T13+T7+T5	90720
submats_7_T24.pbz2	T24+T13+T8+T4	40320
submats_7_T24.pbz2	T24+T13+T8+T5	40320
submats_7_T24.pbz2	T24+T14+T6+T4	12600
submats_7_T24.pbz2	T24+T14+T7+T4	25200
submats_7_T24.pbz2	T24+T14+T7+T5	37800
submats_7_T24.pbz2	T24+T14+T8+T4	12600
submats_7_T24.pbz2	T24+T14+T8+T5	12600
submats_7_T24.pbz2	T24+T9+T6+T4	5040
submats_7_T25.pbz2	T25+T10+T6+T4	5040
submats_7_T25.pbz2	T25+T10+T7+T4	5040
submats_7_T25.pbz2	T25+T10+T7+T5	7560
submats_7_T25.pbz2	T25+T11+T6+T4	2520
submats_7_T25.pbz2	T25+T11+T7+T4	10080
submats_7_T25.pbz2	T25+T11+T7+T5	15120
submats_7_T25.pbz2	T25+T12+T6+T4	2520
submats_7_T25.pbz2	T25+T12+T7+T4	5040
submats_7_T25.pbz2	T25+T12+T7+T5	7560
submats_7_T25.pbz2	T25+T12+T8+T4	7560
submats_7_T25.pbz2	T25+T12+T8+T5	7560
submats_7_T25.pbz2	T25+T13+T6+T4	5040
submats_7_T25.pbz2	T25+T13+T7+T4	15120
submats_7_T25.pbz2	T25+T13+T7+T5	22680
submats_7_T25.pbz2	T25+T13+T8+T4	10080
submats_7_T25.pbz2	T25+T13+T8+T5	10080
submats_7_T25.pbz2	T25+T14+T6+T4	2520
submats_7_T25.pbz2	T25+T14+T7+T4	5040
submats_7_T25.pbz2	T25+T14+T7+T5	7560
submats_7_T25.pbz2	T25+T14+T8+T4	2520
submats_7_T25.pbz2	T25+T14+T8+T5	2520
submats_7_T25.pbz2	T25+T9+T6+T4	2520
submats_8_T26.pbz2	T26+T15+T9+T6+T4	20160
submats_8_T27.pbz2	T27+T15+T9+T6+T4	40320
submats_8_T27.pbz2	T27+T16+T10+T6+T4	40320
submats_8_T27.pbz2	T27+T16+T10+T7+T4	40320
submats_8_T27.pbz2	T27+T16+T10+T7+T5	60480

Continued on next page

Table S2 – continued from previous page

File name	Tree sequence	# matrices
submats_8_T27.pbz2	T27+T16+T9+T6+T4	40320
submats_8_T28.pbz2	T28+T15+T9+T6+T4	40320
submats_8_T28.pbz2	T28+T16+T10+T6+T4	80640
submats_8_T28.pbz2	T28+T16+T10+T7+T4	80640
submats_8_T28.pbz2	T28+T16+T10+T7+T5	120960
submats_8_T28.pbz2	T28+T16+T9+T6+T4	80640
submats_8_T28.pbz2	T28+T17+T10+T6+T4	80640
submats_8_T28.pbz2	T28+T17+T10+T7+T4	80640
submats_8_T28.pbz2	T28+T17+T10+T7+T5	120960
submats_8_T28.pbz2	T28+T17+T11+T6+T4	40320
submats_8_T28.pbz2	T28+T17+T11+T7+T4	161280
submats_8_T28.pbz2	T28+T17+T11+T7+T5	241920
submats_8_T28.pbz2	T28+T17+T9+T6+T4	40320
submats_8_T29.pbz2	T29+T15+T9+T6+T4	20160
submats_8_T29.pbz2	T29+T17+T10+T6+T4	80640
submats_8_T29.pbz2	T29+T17+T10+T7+T4	80640
submats_8_T29.pbz2	T29+T17+T10+T7+T5	120960
submats_8_T29.pbz2	T29+T17+T11+T6+T4	40320
submats_8_T29.pbz2	T29+T17+T11+T7+T4	161280
submats_8_T29.pbz2	T29+T17+T11+T7+T5	241920
submats_8_T29.pbz2	T29+T17+T9+T6+T4	40320
submats_8_T30.pbz2	T30+T17+T10+T6+T4	80640
submats_8_T30.pbz2	T30+T17+T10+T7+T4	80640
submats_8_T30.pbz2	T30+T17+T10+T7+T5	120960
submats_8_T30.pbz2	T30+T17+T11+T6+T4	40320
submats_8_T30.pbz2	T30+T17+T11+T7+T4	161280
submats_8_T30.pbz2	T30+T17+T11+T7+T5	241920
submats_8_T30.pbz2	T30+T17+T9+T6+T4	40320
submats_8_T30.pbz2	T30+T18+T11+T6+T4	60480
submats_8_T30.pbz2	T30+T18+T11+T7+T4	241920
submats_8_T30.pbz2	T30+T18+T11+T7+T5	362880
submats_8_T31.pbz2	T31+T17+T10+T6+T4	80640
submats_8_T31.pbz2	T31+T17+T10+T7+T4	80640
submats_8_T31.pbz2	T31+T17+T10+T7+T5	120960
submats_8_T31.pbz2	T31+T17+T11+T6+T4	40320
submats_8_T31.pbz2	T31+T17+T11+T7+T4	161280
submats_8_T31.pbz2	T31+T17+T11+T7+T5	241920
submats_8_T31.pbz2	T31+T17+T9+T6+T4	40320
submats_8_T31.pbz2	T31+T18+T11+T6+T4	60480
submats_8_T31.pbz2	T31+T18+T11+T7+T4	241920
submats_8_T31.pbz2	T31+T18+T11+T7+T5	362880
submats_8_T31.pbz2	T31+T20+T10+T6+T4	120960
submats_8_T31.pbz2	T31+T20+T10+T7+T4	120960

Continued on next page

Table S2 – continued from previous page

File name	Tree sequence	# matrices
submats_8_T31.pbz2	T31+T20+T10+T7+T5	181440
submats_8_T31.pbz2	T31+T20+T11+T6+T4	40320
submats_8_T31.pbz2	T31+T20+T11+T7+T4	161280
submats_8_T31.pbz2	T31+T20+T11+T7+T5	241920
submats_8_T31.pbz2	T31+T20+T12+T6+T4	80640
submats_8_T31.pbz2	T31+T20+T12+T7+T4	161280
submats_8_T31.pbz2	T31+T20+T12+T7+T5	241920
submats_8_T31.pbz2	T31+T20+T12+T8+T4	241920
submats_8_T31.pbz2	T31+T20+T12+T8+T5	241920
submats_8_T31.pbz2	T31+T20+T13+T6+T4	120960
submats_8_T31.pbz2	T31+T20+T13+T7+T4	362880
submats_8_T31.pbz2	T31+T20+T13+T7+T5	544320
submats_8_T31.pbz2	T31+T20+T13+T8+T4	241920
submats_8_T31.pbz2	T31+T20+T13+T8+T5	241920
submats_8_T31.pbz2	T31+T20+T9+T6+T4	40320
submats_8_T31.pbz2	T31+T22+T10+T6+T4	80640
submats_8_T31.pbz2	T31+T22+T10+T7+T4	80640
submats_8_T31.pbz2	T31+T22+T10+T7+T5	120960
submats_8_T31.pbz2	T31+T22+T11+T6+T4	60480
submats_8_T31.pbz2	T31+T22+T11+T7+T4	241920
submats_8_T31.pbz2	T31+T22+T11+T7+T5	362880
submats_8_T31.pbz2	T31+T22+T12+T6+T4	80640
submats_8_T31.pbz2	T31+T22+T12+T7+T4	161280
submats_8_T31.pbz2	T31+T22+T12+T7+T5	241920
submats_8_T31.pbz2	T31+T22+T12+T8+T4	241920
submats_8_T31.pbz2	T31+T22+T12+T8+T5	241920
submats_8_T31.pbz2	T31+T22+T13+T6+T4	120960
submats_8_T31.pbz2	T31+T22+T13+T7+T4	362880
submats_8_T31.pbz2	T31+T22+T13+T7+T5	544320
submats_8_T31.pbz2	T31+T22+T13+T8+T4	241920
submats_8_T31.pbz2	T31+T22+T13+T8+T5	241920
submats_8_T31.pbz2	T31+T22+T9+T6+T4	20160
submats_8_T32.pbz2	T32+T15+T9+T6+T4	20160
submats_8_T32.pbz2	T32+T16+T10+T6+T4	40320
submats_8_T32.pbz2	T32+T16+T10+T7+T4	40320
submats_8_T32.pbz2	T32+T16+T10+T7+T5	60480
submats_8_T32.pbz2	T32+T16+T9+T6+T4	40320
submats_8_T32.pbz2	T32+T19+T10+T6+T4	40320
submats_8_T32.pbz2	T32+T19+T10+T7+T4	40320
submats_8_T32.pbz2	T32+T19+T10+T7+T5	60480
submats_8_T32.pbz2	T32+T19+T12+T6+T4	20160
submats_8_T32.pbz2	T32+T19+T12+T7+T4	40320
submats_8_T32.pbz2	T32+T19+T12+T7+T5	60480

Continued on next page

Table S2 – continued from previous page

File name	Tree sequence	# matrices
submats_8_T32.pbz2	T32+T19+T12+T8+T4	60480
submats_8_T32.pbz2	T32+T19+T12+T8+T5	60480
submats_8_T32.pbz2	T32+T19+T9+T6+T4	20160
submats_8_T33.pbz2	T33+T15+T9+T6+T4	40320
submats_8_T33.pbz2	T33+T16+T10+T6+T4	120960
submats_8_T33.pbz2	T33+T16+T10+T7+T4	120960
submats_8_T33.pbz2	T33+T16+T10+T7+T5	181440
submats_8_T33.pbz2	T33+T16+T9+T6+T4	120960
submats_8_T33.pbz2	T33+T17+T10+T6+T4	80640
submats_8_T33.pbz2	T33+T17+T10+T7+T4	80640
submats_8_T33.pbz2	T33+T17+T10+T7+T5	120960
submats_8_T33.pbz2	T33+T17+T11+T6+T4	40320
submats_8_T33.pbz2	T33+T17+T11+T7+T4	161280
submats_8_T33.pbz2	T33+T17+T11+T7+T5	241920
submats_8_T33.pbz2	T33+T17+T9+T6+T4	40320
submats_8_T33.pbz2	T33+T19+T10+T6+T4	161280
submats_8_T33.pbz2	T33+T19+T10+T7+T4	161280
submats_8_T33.pbz2	T33+T19+T10+T7+T5	241920
submats_8_T33.pbz2	T33+T19+T12+T6+T4	80640
submats_8_T33.pbz2	T33+T19+T12+T7+T4	161280
submats_8_T33.pbz2	T33+T19+T12+T7+T5	241920
submats_8_T33.pbz2	T33+T19+T12+T8+T4	241920
submats_8_T33.pbz2	T33+T19+T12+T8+T5	241920
submats_8_T33.pbz2	T33+T19+T9+T6+T4	80640
submats_8_T33.pbz2	T33+T20+T10+T6+T4	120960
submats_8_T33.pbz2	T33+T20+T10+T7+T4	120960
submats_8_T33.pbz2	T33+T20+T10+T7+T5	181440
submats_8_T33.pbz2	T33+T20+T11+T6+T4	40320
submats_8_T33.pbz2	T33+T20+T11+T7+T4	161280
submats_8_T33.pbz2	T33+T20+T11+T7+T5	241920
submats_8_T33.pbz2	T33+T20+T12+T6+T4	80640
submats_8_T33.pbz2	T33+T20+T12+T7+T4	161280
submats_8_T33.pbz2	T33+T20+T12+T7+T5	241920
submats_8_T33.pbz2	T33+T20+T12+T8+T4	241920
submats_8_T33.pbz2	T33+T20+T12+T8+T5	241920
submats_8_T33.pbz2	T33+T20+T13+T6+T4	120960
submats_8_T33.pbz2	T33+T20+T13+T7+T4	362880
submats_8_T33.pbz2	T33+T20+T13+T7+T5	544320
submats_8_T33.pbz2	T33+T20+T13+T8+T4	241920
submats_8_T33.pbz2	T33+T20+T13+T8+T5	241920
submats_8_T33.pbz2	T33+T20+T9+T6+T4	40320
submats_8_T34.pbz2	T34+T15+T9+T6+T4	20160
submats_8_T34.pbz2	T34+T16+T10+T6+T4	80640

Continued on next page

Table S2 – continued from previous page

File name	Tree sequence	# matrices
submats_8_T34.pbz2	T34+T16+T10+T7+T4	80640
submats_8_T34.pbz2	T34+T16+T10+T7+T5	120960
submats_8_T34.pbz2	T34+T16+T9+T6+T4	80640
submats_8_T34.pbz2	T34+T17+T10+T6+T4	80640
submats_8_T34.pbz2	T34+T17+T10+T7+T4	80640
submats_8_T34.pbz2	T34+T17+T10+T7+T5	120960
submats_8_T34.pbz2	T34+T17+T11+T6+T4	40320
submats_8_T34.pbz2	T34+T17+T11+T7+T4	161280
submats_8_T34.pbz2	T34+T17+T11+T7+T5	241920
submats_8_T34.pbz2	T34+T17+T9+T6+T4	40320
submats_8_T34.pbz2	T34+T19+T10+T6+T4	161280
submats_8_T34.pbz2	T34+T19+T10+T7+T4	161280
submats_8_T34.pbz2	T34+T19+T10+T7+T5	241920
submats_8_T34.pbz2	T34+T19+T12+T6+T4	80640
submats_8_T34.pbz2	T34+T19+T12+T7+T4	161280
submats_8_T34.pbz2	T34+T19+T12+T7+T5	241920
submats_8_T34.pbz2	T34+T19+T12+T8+T4	241920
submats_8_T34.pbz2	T34+T19+T12+T8+T5	241920
submats_8_T34.pbz2	T34+T19+T9+T6+T4	80640
submats_8_T34.pbz2	T34+T20+T10+T6+T4	241920
submats_8_T34.pbz2	T34+T20+T10+T7+T4	241920
submats_8_T34.pbz2	T34+T20+T10+T7+T5	362880
submats_8_T34.pbz2	T34+T20+T11+T6+T4	80640
submats_8_T34.pbz2	T34+T20+T11+T7+T4	322560
submats_8_T34.pbz2	T34+T20+T11+T7+T5	483840
submats_8_T34.pbz2	T34+T20+T12+T6+T4	161280
submats_8_T34.pbz2	T34+T20+T12+T7+T4	322560
submats_8_T34.pbz2	T34+T20+T12+T7+T5	483840
submats_8_T34.pbz2	T34+T20+T12+T8+T4	483840
submats_8_T34.pbz2	T34+T20+T12+T8+T5	483840
submats_8_T34.pbz2	T34+T20+T13+T6+T4	241920
submats_8_T34.pbz2	T34+T20+T13+T7+T4	725760
submats_8_T34.pbz2	T34+T20+T13+T7+T5	1088640
submats_8_T34.pbz2	T34+T20+T13+T8+T4	483840
submats_8_T34.pbz2	T34+T20+T13+T8+T5	483840
submats_8_T34.pbz2	T34+T20+T9+T6+T4	80640
submats_8_T34.pbz2	T34+T22+T10+T6+T4	161280
submats_8_T34.pbz2	T34+T22+T10+T7+T4	161280
submats_8_T34.pbz2	T34+T22+T10+T7+T5	241920
submats_8_T34.pbz2	T34+T22+T11+T6+T4	120960
submats_8_T34.pbz2	T34+T22+T11+T7+T4	483840
submats_8_T34.pbz2	T34+T22+T11+T7+T5	725760
submats_8_T34.pbz2	T34+T22+T12+T6+T4	161280

Continued on next page

Table S2 – continued from previous page

File name	Tree sequence	# matrices
submats_8_T34.pbz2	T34+T22+T12+T7+T4	322560
submats_8_T34.pbz2	T34+T22+T12+T7+T5	483840
submats_8_T34.pbz2	T34+T22+T12+T8+T4	483840
submats_8_T34.pbz2	T34+T22+T12+T8+T5	483840
submats_8_T34.pbz2	T34+T22+T13+T6+T4	241920
submats_8_T34.pbz2	T34+T22+T13+T7+T4	725760
submats_8_T34.pbz2	T34+T22+T13+T7+T5	1088640
submats_8_T34.pbz2	T34+T22+T13+T8+T4	483840
submats_8_T34.pbz2	T34+T22+T13+T8+T5	483840
submats_8_T34.pbz2	T34+T22+T9+T6+T4	40320
submats_8_T35.pbz2	T35+T15+T9+T6+T4	40320
submats_8_T35.pbz2	T35+T16+T10+T6+T4	40320
submats_8_T35.pbz2	T35+T16+T10+T7+T4	40320
submats_8_T35.pbz2	T35+T16+T10+T7+T5	60480
submats_8_T35.pbz2	T35+T16+T9+T6+T4	40320
submats_8_T35.pbz2	T35+T17+T10+T6+T4	161280
submats_8_T35.pbz2	T35+T17+T10+T7+T4	161280
submats_8_T35.pbz2	T35+T17+T10+T7+T5	241920
submats_8_T35.pbz2	T35+T17+T11+T6+T4	80640
submats_8_T35.pbz2	T35+T17+T11+T7+T4	322560
submats_8_T35.pbz2	T35+T17+T11+T7+T5	483840
submats_8_T35.pbz2	T35+T17+T9+T6+T4	80640
submats_8_T35.pbz2	T35+T20+T10+T6+T4	120960
submats_8_T35.pbz2	T35+T20+T10+T7+T4	120960
submats_8_T35.pbz2	T35+T20+T10+T7+T5	181440
submats_8_T35.pbz2	T35+T20+T11+T6+T4	40320
submats_8_T35.pbz2	T35+T20+T11+T7+T4	161280
submats_8_T35.pbz2	T35+T20+T11+T7+T5	241920
submats_8_T35.pbz2	T35+T20+T12+T6+T4	80640
submats_8_T35.pbz2	T35+T20+T12+T7+T4	161280
submats_8_T35.pbz2	T35+T20+T12+T7+T5	241920
submats_8_T35.pbz2	T35+T20+T12+T8+T4	241920
submats_8_T35.pbz2	T35+T20+T12+T8+T5	241920
submats_8_T35.pbz2	T35+T20+T13+T6+T4	120960
submats_8_T35.pbz2	T35+T20+T13+T7+T4	362880
submats_8_T35.pbz2	T35+T20+T13+T7+T5	544320
submats_8_T35.pbz2	T35+T20+T13+T8+T4	241920
submats_8_T35.pbz2	T35+T20+T13+T8+T5	241920
submats_8_T35.pbz2	T35+T20+T9+T6+T4	40320
submats_8_T35.pbz2	T35+T21+T10+T6+T4	120960
submats_8_T35.pbz2	T35+T21+T10+T7+T4	120960
submats_8_T35.pbz2	T35+T21+T10+T7+T5	181440
submats_8_T35.pbz2	T35+T21+T11+T6+T4	120960

Continued on next page

Table S2 – continued from previous page

File name	Tree sequence	# matrices
submats_8_T35.pbz2	T35+T21+T11+T7+T4	483840
submats_8_T35.pbz2	T35+T21+T11+T7+T5	725760
submats_8_T35.pbz2	T35+T21+T13+T6+T4	120960
submats_8_T35.pbz2	T35+T21+T13+T7+T4	362880
submats_8_T35.pbz2	T35+T21+T13+T7+T5	544320
submats_8_T35.pbz2	T35+T21+T13+T8+T4	241920
submats_8_T35.pbz2	T35+T21+T13+T8+T5	241920
submats_8_T35.pbz2	T35+T21+T9+T6+T4	120960
submats_8_T36.pbz2	T36+T15+T9+T6+T4	20160
submats_8_T36.pbz2	T36+T16+T10+T6+T4	40320
submats_8_T36.pbz2	T36+T16+T10+T7+T4	40320
submats_8_T36.pbz2	T36+T16+T10+T7+T5	60480
submats_8_T36.pbz2	T36+T16+T9+T6+T4	40320
submats_8_T36.pbz2	T36+T17+T10+T6+T4	80640
submats_8_T36.pbz2	T36+T17+T10+T7+T4	80640
submats_8_T36.pbz2	T36+T17+T10+T7+T5	120960
submats_8_T36.pbz2	T36+T17+T11+T6+T4	40320
submats_8_T36.pbz2	T36+T17+T11+T7+T4	161280
submats_8_T36.pbz2	T36+T17+T11+T7+T5	241920
submats_8_T36.pbz2	T36+T17+T9+T6+T4	40320
submats_8_T36.pbz2	T36+T19+T10+T6+T4	40320
submats_8_T36.pbz2	T36+T19+T10+T7+T4	40320
submats_8_T36.pbz2	T36+T19+T10+T7+T5	60480
submats_8_T36.pbz2	T36+T19+T12+T6+T4	20160
submats_8_T36.pbz2	T36+T19+T12+T7+T4	40320
submats_8_T36.pbz2	T36+T19+T12+T7+T5	60480
submats_8_T36.pbz2	T36+T19+T12+T8+T4	60480
submats_8_T36.pbz2	T36+T19+T12+T8+T5	60480
submats_8_T36.pbz2	T36+T19+T9+T6+T4	20160
submats_8_T36.pbz2	T36+T20+T10+T6+T4	120960
submats_8_T36.pbz2	T36+T20+T10+T7+T4	120960
submats_8_T36.pbz2	T36+T20+T10+T7+T5	181440
submats_8_T36.pbz2	T36+T20+T11+T6+T4	40320
submats_8_T36.pbz2	T36+T20+T11+T7+T4	161280
submats_8_T36.pbz2	T36+T20+T11+T7+T5	241920
submats_8_T36.pbz2	T36+T20+T12+T6+T4	80640
submats_8_T36.pbz2	T36+T20+T12+T7+T4	161280
submats_8_T36.pbz2	T36+T20+T12+T7+T5	241920
submats_8_T36.pbz2	T36+T20+T12+T8+T4	241920
submats_8_T36.pbz2	T36+T20+T12+T8+T5	241920
submats_8_T36.pbz2	T36+T20+T13+T6+T4	120960
submats_8_T36.pbz2	T36+T20+T13+T7+T4	362880
submats_8_T36.pbz2	T36+T20+T13+T7+T5	544320

Continued on next page

Table S2 – continued from previous page

File name	Tree sequence	# matrices
submats_8_T36.pbz2	T36+T20+T13+T8+T4	241920
submats_8_T36.pbz2	T36+T20+T13+T8+T5	241920
submats_8_T36.pbz2	T36+T20+T9+T6+T4	40320
submats_8_T36.pbz2	T36+T21+T10+T6+T4	120960
submats_8_T36.pbz2	T36+T21+T10+T7+T4	120960
submats_8_T36.pbz2	T36+T21+T10+T7+T5	181440
submats_8_T36.pbz2	T36+T21+T11+T6+T4	120960
submats_8_T36.pbz2	T36+T21+T11+T7+T4	483840
submats_8_T36.pbz2	T36+T21+T11+T7+T5	725760
submats_8_T36.pbz2	T36+T21+T13+T6+T4	120960
submats_8_T36.pbz2	T36+T21+T13+T7+T4	362880
submats_8_T36.pbz2	T36+T21+T13+T7+T5	544320
submats_8_T36.pbz2	T36+T21+T13+T8+T4	241920
submats_8_T36.pbz2	T36+T21+T13+T8+T5	241920
submats_8_T36.pbz2	T36+T21+T9+T6+T4	120960
submats_8_T36.pbz2	T36+T23+T10+T6+T4	241920
submats_8_T36.pbz2	T36+T23+T10+T7+T4	241920
submats_8_T36.pbz2	T36+T23+T10+T7+T5	362880
submats_8_T36.pbz2	T36+T23+T11+T6+T4	120960
submats_8_T36.pbz2	T36+T23+T11+T7+T4	483840
submats_8_T36.pbz2	T36+T23+T11+T7+T5	725760
submats_8_T36.pbz2	T36+T23+T12+T6+T4	120960
submats_8_T36.pbz2	T36+T23+T12+T7+T4	241920
submats_8_T36.pbz2	T36+T23+T12+T7+T5	362880
submats_8_T36.pbz2	T36+T23+T12+T8+T4	362880
submats_8_T36.pbz2	T36+T23+T12+T8+T5	362880
submats_8_T36.pbz2	T36+T23+T13+T6+T4	483840
submats_8_T36.pbz2	T36+T23+T13+T7+T4	1451520
submats_8_T36.pbz2	T36+T23+T13+T7+T5	2177280
submats_8_T36.pbz2	T36+T23+T13+T8+T4	967680
submats_8_T36.pbz2	T36+T23+T13+T8+T5	967680
submats_8_T36.pbz2	T36+T23+T14+T6+T4	604800
submats_8_T36.pbz2	T36+T23+T14+T7+T4	1209600
submats_8_T36.pbz2	T36+T23+T14+T7+T5	1814400
submats_8_T36.pbz2	T36+T23+T14+T8+T4	604800
submats_8_T36.pbz2	T36+T23+T14+T8+T5	604800
submats_8_T36.pbz2	T36+T23+T9+T6+T4	120960
submats_8_T37.pbz2	T37+T17+T10+T6+T4	80640
submats_8_T37.pbz2	T37+T17+T10+T7+T4	80640
submats_8_T37.pbz2	T37+T17+T10+T7+T5	120960
submats_8_T37.pbz2	T37+T17+T11+T6+T4	40320
submats_8_T37.pbz2	T37+T17+T11+T7+T4	161280
submats_8_T37.pbz2	T37+T17+T11+T7+T5	241920

Continued on next page

Table S2 – continued from previous page

File name	Tree sequence	# matrices
submats_8_T37.pbz2	T37+T17+T9+T6+T4	40320
submats_8_T37.pbz2	T37+T18+T11+T6+T4	20160
submats_8_T37.pbz2	T37+T18+T11+T7+T4	80640
submats_8_T37.pbz2	T37+T18+T11+T7+T5	120960
submats_8_T37.pbz2	T37+T20+T10+T6+T4	241920
submats_8_T37.pbz2	T37+T20+T10+T7+T4	241920
submats_8_T37.pbz2	T37+T20+T10+T7+T5	362880
submats_8_T37.pbz2	T37+T20+T11+T6+T4	80640
submats_8_T37.pbz2	T37+T20+T11+T7+T4	322560
submats_8_T37.pbz2	T37+T20+T11+T7+T5	483840
submats_8_T37.pbz2	T37+T20+T12+T6+T4	161280
submats_8_T37.pbz2	T37+T20+T12+T7+T4	322560
submats_8_T37.pbz2	T37+T20+T12+T7+T5	483840
submats_8_T37.pbz2	T37+T20+T12+T8+T4	483840
submats_8_T37.pbz2	T37+T20+T12+T8+T5	483840
submats_8_T37.pbz2	T37+T20+T13+T6+T4	241920
submats_8_T37.pbz2	T37+T20+T13+T7+T4	725760
submats_8_T37.pbz2	T37+T20+T13+T7+T5	1088640
submats_8_T37.pbz2	T37+T20+T13+T8+T4	483840
submats_8_T37.pbz2	T37+T20+T13+T8+T5	483840
submats_8_T37.pbz2	T37+T20+T9+T6+T4	80640
submats_8_T37.pbz2	T37+T22+T10+T6+T4	161280
submats_8_T37.pbz2	T37+T22+T10+T7+T4	161280
submats_8_T37.pbz2	T37+T22+T10+T7+T5	241920
submats_8_T37.pbz2	T37+T22+T11+T6+T4	120960
submats_8_T37.pbz2	T37+T22+T11+T7+T4	483840
submats_8_T37.pbz2	T37+T22+T11+T7+T5	725760
submats_8_T37.pbz2	T37+T22+T12+T6+T4	161280
submats_8_T37.pbz2	T37+T22+T12+T7+T4	322560
submats_8_T37.pbz2	T37+T22+T12+T7+T5	483840
submats_8_T37.pbz2	T37+T22+T12+T8+T4	483840
submats_8_T37.pbz2	T37+T22+T12+T8+T5	483840
submats_8_T37.pbz2	T37+T22+T13+T6+T4	241920
submats_8_T37.pbz2	T37+T22+T13+T7+T4	725760
submats_8_T37.pbz2	T37+T22+T13+T7+T5	1088640
submats_8_T37.pbz2	T37+T22+T13+T8+T4	483840
submats_8_T37.pbz2	T37+T22+T13+T8+T5	483840
submats_8_T37.pbz2	T37+T22+T9+T6+T4	40320
submats_8_T38.pbz2	T38+T15+T9+T6+T4	40320
submats_8_T38.pbz2	T38+T16+T10+T6+T4	120960
submats_8_T38.pbz2	T38+T16+T10+T7+T4	120960
submats_8_T38.pbz2	T38+T16+T10+T7+T5	181440
submats_8_T38.pbz2	T38+T16+T9+T6+T4	120960

Continued on next page

Table S2 – continued from previous page

File name	Tree sequence	# matrices
submats_8_T38.pbz2	T38+T17+T10+T6+T4	241920
submats_8_T38.pbz2	T38+T17+T10+T7+T4	241920
submats_8_T38.pbz2	T38+T17+T10+T7+T5	362880
submats_8_T38.pbz2	T38+T17+T11+T6+T4	120960
submats_8_T38.pbz2	T38+T17+T11+T7+T4	483840
submats_8_T38.pbz2	T38+T17+T11+T7+T5	725760
submats_8_T38.pbz2	T38+T17+T9+T6+T4	120960
submats_8_T38.pbz2	T38+T18+T11+T6+T4	120960
submats_8_T38.pbz2	T38+T18+T11+T7+T4	483840
submats_8_T38.pbz2	T38+T18+T11+T7+T5	725760
submats_8_T38.pbz2	T38+T19+T10+T6+T4	161280
submats_8_T38.pbz2	T38+T19+T10+T7+T4	161280
submats_8_T38.pbz2	T38+T19+T10+T7+T5	241920
submats_8_T38.pbz2	T38+T19+T12+T6+T4	80640
submats_8_T38.pbz2	T38+T19+T12+T7+T4	161280
submats_8_T38.pbz2	T38+T19+T12+T7+T5	241920
submats_8_T38.pbz2	T38+T19+T12+T8+T4	241920
submats_8_T38.pbz2	T38+T19+T12+T8+T5	241920
submats_8_T38.pbz2	T38+T19+T9+T6+T4	80640
submats_8_T38.pbz2	T38+T20+T10+T6+T4	483840
submats_8_T38.pbz2	T38+T20+T10+T7+T4	483840
submats_8_T38.pbz2	T38+T20+T10+T7+T5	725760
submats_8_T38.pbz2	T38+T20+T11+T6+T4	161280
submats_8_T38.pbz2	T38+T20+T11+T7+T4	645120
submats_8_T38.pbz2	T38+T20+T11+T7+T5	967680
submats_8_T38.pbz2	T38+T20+T12+T6+T4	322560
submats_8_T38.pbz2	T38+T20+T12+T7+T4	645120
submats_8_T38.pbz2	T38+T20+T12+T7+T5	967680
submats_8_T38.pbz2	T38+T20+T12+T8+T4	967680
submats_8_T38.pbz2	T38+T20+T12+T8+T5	967680
submats_8_T38.pbz2	T38+T20+T13+T6+T4	483840
submats_8_T38.pbz2	T38+T20+T13+T7+T4	1451520
submats_8_T38.pbz2	T38+T20+T13+T7+T5	2177280
submats_8_T38.pbz2	T38+T20+T13+T8+T4	967680
submats_8_T38.pbz2	T38+T20+T13+T8+T5	967680
submats_8_T38.pbz2	T38+T20+T9+T6+T4	161280
submats_8_T38.pbz2	T38+T21+T10+T6+T4	241920
submats_8_T38.pbz2	T38+T21+T10+T7+T4	241920
submats_8_T38.pbz2	T38+T21+T10+T7+T5	362880
submats_8_T38.pbz2	T38+T21+T11+T6+T4	241920
submats_8_T38.pbz2	T38+T21+T11+T7+T4	967680
submats_8_T38.pbz2	T38+T21+T11+T7+T5	1451520
submats_8_T38.pbz2	T38+T21+T13+T6+T4	241920

Continued on next page

Table S2 – continued from previous page

File name	Tree sequence	# matrices
submats_8_T38.pbz2	T38+T21+T13+T7+T4	725760
submats_8_T38.pbz2	T38+T21+T13+T7+T5	1088640
submats_8_T38.pbz2	T38+T21+T13+T8+T4	483840
submats_8_T38.pbz2	T38+T21+T13+T8+T5	483840
submats_8_T38.pbz2	T38+T21+T9+T6+T4	241920
submats_8_T38.pbz2	T38+T22+T10+T6+T4	322560
submats_8_T38.pbz2	T38+T22+T10+T7+T4	322560
submats_8_T38.pbz2	T38+T22+T10+T7+T5	483840
submats_8_T38.pbz2	T38+T22+T11+T6+T4	241920
submats_8_T38.pbz2	T38+T22+T11+T7+T4	967680
submats_8_T38.pbz2	T38+T22+T11+T7+T5	1451520
submats_8_T38.pbz2	T38+T22+T12+T6+T4	322560
submats_8_T38.pbz2	T38+T22+T12+T7+T4	645120
submats_8_T38.pbz2	T38+T22+T12+T7+T5	967680
submats_8_T38.pbz2	T38+T22+T12+T8+T4	967680
submats_8_T38.pbz2	T38+T22+T12+T8+T5	967680
submats_8_T38.pbz2	T38+T22+T13+T6+T4	483840
submats_8_T38.pbz2	T38+T22+T13+T7+T4	1451520
submats_8_T38.pbz2	T38+T22+T13+T7+T5	2177280
submats_8_T38.pbz2	T38+T22+T13+T8+T4	967680
submats_8_T38.pbz2	T38+T22+T13+T8+T5	967680
submats_8_T38.pbz2	T38+T22+T9+T6+T4	80640
submats_8_T38.pbz2	T38+T23+T10+T6+T4	725760
submats_8_T38.pbz2	T38+T23+T10+T7+T4	725760
submats_8_T38.pbz2	T38+T23+T10+T7+T5	1088640
submats_8_T38.pbz2	T38+T23+T11+T6+T4	362880
submats_8_T38.pbz2	T38+T23+T11+T7+T4	1451520
submats_8_T38.pbz2	T38+T23+T11+T7+T5	2177280
submats_8_T38.pbz2	T38+T23+T12+T6+T4	362880
submats_8_T38.pbz2	T38+T23+T12+T7+T4	725760
submats_8_T38.pbz2	T38+T23+T12+T7+T5	1088640
submats_8_T38.pbz2	T38+T23+T12+T8+T4	1088640
submats_8_T38.pbz2	T38+T23+T12+T8+T5	1088640
submats_8_T38.pbz2	T38+T23+T13+T6+T4	1451520
submats_8_T38.pbz2	T38+T23+T13+T7+T4	4354560
submats_8_T38.pbz2	T38+T23+T13+T7+T5	6531840
submats_8_T38.pbz2	T38+T23+T13+T8+T4	2903040
submats_8_T38.pbz2	T38+T23+T13+T8+T5	2903040
submats_8_T38.pbz2	T38+T23+T14+T6+T4	1814400
submats_8_T38.pbz2	T38+T23+T14+T7+T4	3628800
submats_8_T38.pbz2	T38+T23+T14+T7+T5	5443200
submats_8_T38.pbz2	T38+T23+T14+T8+T4	1814400
submats_8_T38.pbz2	T38+T23+T14+T8+T5	1814400

Continued on next page

Table S2 – continued from previous page

File name	Tree sequence	# matrices
submats_8_T38.pbz2	T38+T23+T9+T6+T4	362880
submats_8_T38.pbz2	T38+T24+T10+T6+T4	725760
submats_8_T38.pbz2	T38+T24+T10+T7+T4	725760
submats_8_T38.pbz2	T38+T24+T10+T7+T5	1088640
submats_8_T38.pbz2	T38+T24+T11+T6+T4	362880
submats_8_T38.pbz2	T38+T24+T11+T7+T4	1451520
submats_8_T38.pbz2	T38+T24+T11+T7+T5	2177280
submats_8_T38.pbz2	T38+T24+T12+T6+T4	483840
submats_8_T38.pbz2	T38+T24+T12+T7+T4	967680
submats_8_T38.pbz2	T38+T24+T12+T7+T5	1451520
submats_8_T38.pbz2	T38+T24+T12+T8+T4	1451520
submats_8_T38.pbz2	T38+T24+T12+T8+T5	1451520
submats_8_T38.pbz2	T38+T24+T13+T6+T4	967680
submats_8_T38.pbz2	T38+T24+T13+T7+T4	2903040
submats_8_T38.pbz2	T38+T24+T13+T7+T5	4354560
submats_8_T38.pbz2	T38+T24+T13+T8+T4	1935360
submats_8_T38.pbz2	T38+T24+T13+T8+T5	1935360
submats_8_T38.pbz2	T38+T24+T14+T6+T4	604800
submats_8_T38.pbz2	T38+T24+T14+T7+T4	1209600
submats_8_T38.pbz2	T38+T24+T14+T7+T5	1814400
submats_8_T38.pbz2	T38+T24+T14+T8+T4	604800
submats_8_T38.pbz2	T38+T24+T14+T8+T5	604800
submats_8_T38.pbz2	T38+T24+T9+T6+T4	241920
submats_8_T39.pbz2	T39+T15+T9+T6+T4	40320
submats_8_T39.pbz2	T39+T16+T10+T6+T4	40320
submats_8_T39.pbz2	T39+T16+T10+T7+T4	40320
submats_8_T39.pbz2	T39+T16+T10+T7+T5	60480
submats_8_T39.pbz2	T39+T16+T9+T6+T4	40320
submats_8_T39.pbz2	T39+T17+T10+T6+T4	80640
submats_8_T39.pbz2	T39+T17+T10+T7+T4	80640
submats_8_T39.pbz2	T39+T17+T10+T7+T5	120960
submats_8_T39.pbz2	T39+T17+T11+T6+T4	40320
submats_8_T39.pbz2	T39+T17+T11+T7+T4	161280
submats_8_T39.pbz2	T39+T17+T11+T7+T5	241920
submats_8_T39.pbz2	T39+T17+T9+T6+T4	40320
submats_8_T39.pbz2	T39+T21+T10+T6+T4	40320
submats_8_T39.pbz2	T39+T21+T10+T7+T4	40320
submats_8_T39.pbz2	T39+T21+T10+T7+T5	60480
submats_8_T39.pbz2	T39+T21+T11+T6+T4	40320
submats_8_T39.pbz2	T39+T21+T11+T7+T4	161280
submats_8_T39.pbz2	T39+T21+T11+T7+T5	241920
submats_8_T39.pbz2	T39+T21+T13+T6+T4	40320
submats_8_T39.pbz2	T39+T21+T13+T7+T4	120960

Continued on next page

Table S2 – continued from previous page

File name	Tree sequence	# matrices
submats_8_T39.pbz2	T39+T21+T13+T7+T5	181440
submats_8_T39.pbz2	T39+T21+T13+T8+T4	80640
submats_8_T39.pbz2	T39+T21+T13+T8+T5	80640
submats_8_T39.pbz2	T39+T21+T9+T6+T4	40320
submats_8_T40.pbz2	T40+T15+T9+T6+T4	40320
submats_8_T40.pbz2	T40+T16+T10+T6+T4	80640
submats_8_T40.pbz2	T40+T16+T10+T7+T4	80640
submats_8_T40.pbz2	T40+T16+T10+T7+T5	120960
submats_8_T40.pbz2	T40+T16+T9+T6+T4	80640
submats_8_T40.pbz2	T40+T17+T10+T6+T4	241920
submats_8_T40.pbz2	T40+T17+T10+T7+T4	241920
submats_8_T40.pbz2	T40+T17+T10+T7+T5	362880
submats_8_T40.pbz2	T40+T17+T11+T6+T4	120960
submats_8_T40.pbz2	T40+T17+T11+T7+T4	483840
submats_8_T40.pbz2	T40+T17+T11+T7+T5	725760
submats_8_T40.pbz2	T40+T17+T9+T6+T4	120960
submats_8_T40.pbz2	T40+T18+T11+T6+T4	120960
submats_8_T40.pbz2	T40+T18+T11+T7+T4	483840
submats_8_T40.pbz2	T40+T18+T11+T7+T5	725760
submats_8_T40.pbz2	T40+T20+T10+T6+T4	241920
submats_8_T40.pbz2	T40+T20+T10+T7+T4	241920
submats_8_T40.pbz2	T40+T20+T10+T7+T5	362880
submats_8_T40.pbz2	T40+T20+T11+T6+T4	80640
submats_8_T40.pbz2	T40+T20+T11+T7+T4	322560
submats_8_T40.pbz2	T40+T20+T11+T7+T5	483840
submats_8_T40.pbz2	T40+T20+T12+T6+T4	161280
submats_8_T40.pbz2	T40+T20+T12+T7+T4	322560
submats_8_T40.pbz2	T40+T20+T12+T7+T5	483840
submats_8_T40.pbz2	T40+T20+T12+T8+T4	483840
submats_8_T40.pbz2	T40+T20+T12+T8+T5	483840
submats_8_T40.pbz2	T40+T20+T13+T6+T4	241920
submats_8_T40.pbz2	T40+T20+T13+T7+T4	725760
submats_8_T40.pbz2	T40+T20+T13+T7+T5	1088640
submats_8_T40.pbz2	T40+T20+T13+T8+T4	483840
submats_8_T40.pbz2	T40+T20+T13+T8+T5	483840
submats_8_T40.pbz2	T40+T20+T9+T6+T4	80640
submats_8_T40.pbz2	T40+T21+T10+T6+T4	120960
submats_8_T40.pbz2	T40+T21+T10+T7+T4	120960
submats_8_T40.pbz2	T40+T21+T10+T7+T5	181440
submats_8_T40.pbz2	T40+T21+T11+T6+T4	120960
submats_8_T40.pbz2	T40+T21+T11+T7+T4	483840
submats_8_T40.pbz2	T40+T21+T11+T7+T5	725760
submats_8_T40.pbz2	T40+T21+T13+T6+T4	120960

Continued on next page

Table S2 – continued from previous page

File name	Tree sequence	# matrices
submats_8_T40.pbz2	T40+T21+T13+T7+T4	362880
submats_8_T40.pbz2	T40+T21+T13+T7+T5	544320
submats_8_T40.pbz2	T40+T21+T13+T8+T4	241920
submats_8_T40.pbz2	T40+T21+T13+T8+T5	241920
submats_8_T40.pbz2	T40+T21+T9+T6+T4	120960
submats_8_T40.pbz2	T40+T22+T10+T6+T4	161280
submats_8_T40.pbz2	T40+T22+T10+T7+T4	161280
submats_8_T40.pbz2	T40+T22+T10+T7+T5	241920
submats_8_T40.pbz2	T40+T22+T11+T6+T4	120960
submats_8_T40.pbz2	T40+T22+T11+T7+T4	483840
submats_8_T40.pbz2	T40+T22+T11+T7+T5	725760
submats_8_T40.pbz2	T40+T22+T12+T6+T4	161280
submats_8_T40.pbz2	T40+T22+T12+T7+T4	322560
submats_8_T40.pbz2	T40+T22+T12+T7+T5	483840
submats_8_T40.pbz2	T40+T22+T12+T8+T4	483840
submats_8_T40.pbz2	T40+T22+T12+T8+T5	483840
submats_8_T40.pbz2	T40+T22+T13+T6+T4	241920
submats_8_T40.pbz2	T40+T22+T13+T7+T4	725760
submats_8_T40.pbz2	T40+T22+T13+T7+T5	1088640
submats_8_T40.pbz2	T40+T22+T13+T8+T4	483840
submats_8_T40.pbz2	T40+T22+T13+T8+T5	483840
submats_8_T40.pbz2	T40+T22+T9+T6+T4	40320
submats_8_T41.pbz2	T41+T15+T9+T6+T4	40320
submats_8_T41.pbz2	T41+T16+T10+T6+T4	80640
submats_8_T41.pbz2	T41+T16+T10+T7+T4	80640
submats_8_T41.pbz2	T41+T16+T10+T7+T5	120960
submats_8_T41.pbz2	T41+T16+T9+T6+T4	80640
submats_8_T41.pbz2	T41+T17+T10+T6+T4	80640
submats_8_T41.pbz2	T41+T17+T10+T7+T4	80640
submats_8_T41.pbz2	T41+T17+T10+T7+T5	120960
submats_8_T41.pbz2	T41+T17+T11+T6+T4	40320
submats_8_T41.pbz2	T41+T17+T11+T7+T4	161280
submats_8_T41.pbz2	T41+T17+T11+T7+T5	241920
submats_8_T41.pbz2	T41+T17+T9+T6+T4	40320
submats_8_T41.pbz2	T41+T19+T10+T6+T4	80640
submats_8_T41.pbz2	T41+T19+T10+T7+T4	80640
submats_8_T41.pbz2	T41+T19+T10+T7+T5	120960
submats_8_T41.pbz2	T41+T19+T12+T6+T4	40320
submats_8_T41.pbz2	T41+T19+T12+T7+T4	80640
submats_8_T41.pbz2	T41+T19+T12+T7+T5	120960
submats_8_T41.pbz2	T41+T19+T12+T8+T4	120960
submats_8_T41.pbz2	T41+T19+T12+T8+T5	120960
submats_8_T41.pbz2	T41+T19+T9+T6+T4	40320

Continued on next page

Table S2 – continued from previous page

File name	Tree sequence	# matrices
submats_8_T41.pbz2	T41+T20+T10+T6+T4	120960
submats_8_T41.pbz2	T41+T20+T10+T7+T4	120960
submats_8_T41.pbz2	T41+T20+T10+T7+T5	181440
submats_8_T41.pbz2	T41+T20+T11+T6+T4	40320
submats_8_T41.pbz2	T41+T20+T11+T7+T4	161280
submats_8_T41.pbz2	T41+T20+T11+T7+T5	241920
submats_8_T41.pbz2	T41+T20+T12+T6+T4	80640
submats_8_T41.pbz2	T41+T20+T12+T7+T4	161280
submats_8_T41.pbz2	T41+T20+T12+T7+T5	241920
submats_8_T41.pbz2	T41+T20+T12+T8+T4	241920
submats_8_T41.pbz2	T41+T20+T12+T8+T5	241920
submats_8_T41.pbz2	T41+T20+T13+T6+T4	120960
submats_8_T41.pbz2	T41+T20+T13+T7+T4	362880
submats_8_T41.pbz2	T41+T20+T13+T7+T5	544320
submats_8_T41.pbz2	T41+T20+T13+T8+T4	241920
submats_8_T41.pbz2	T41+T20+T13+T8+T5	241920
submats_8_T41.pbz2	T41+T20+T9+T6+T4	40320
submats_8_T41.pbz2	T41+T21+T10+T6+T4	40320
submats_8_T41.pbz2	T41+T21+T10+T7+T4	40320
submats_8_T41.pbz2	T41+T21+T10+T7+T5	60480
submats_8_T41.pbz2	T41+T21+T11+T6+T4	40320
submats_8_T41.pbz2	T41+T21+T11+T7+T4	161280
submats_8_T41.pbz2	T41+T21+T11+T7+T5	241920
submats_8_T41.pbz2	T41+T21+T13+T6+T4	40320
submats_8_T41.pbz2	T41+T21+T13+T7+T4	120960
submats_8_T41.pbz2	T41+T21+T13+T7+T5	181440
submats_8_T41.pbz2	T41+T21+T13+T8+T4	80640
submats_8_T41.pbz2	T41+T21+T13+T8+T5	80640
submats_8_T41.pbz2	T41+T21+T9+T6+T4	40320
submats_8_T41.pbz2	T41+T23+T10+T6+T4	80640
submats_8_T41.pbz2	T41+T23+T10+T7+T4	80640
submats_8_T41.pbz2	T41+T23+T10+T7+T5	120960
submats_8_T41.pbz2	T41+T23+T11+T6+T4	40320
submats_8_T41.pbz2	T41+T23+T11+T7+T4	161280
submats_8_T41.pbz2	T41+T23+T11+T7+T5	241920
submats_8_T41.pbz2	T41+T23+T12+T6+T4	40320
submats_8_T41.pbz2	T41+T23+T12+T7+T4	80640
submats_8_T41.pbz2	T41+T23+T12+T7+T5	120960
submats_8_T41.pbz2	T41+T23+T12+T8+T4	120960
submats_8_T41.pbz2	T41+T23+T12+T8+T5	120960
submats_8_T41.pbz2	T41+T23+T13+T6+T4	161280
submats_8_T41.pbz2	T41+T23+T13+T7+T4	483840
submats_8_T41.pbz2	T41+T23+T13+T7+T5	725760

Continued on next page

Table S2 – continued from previous page

File name	Tree sequence	# matrices
submats_8_T41.pbz2	T41+T23+T13+T8+T4	322560
submats_8_T41.pbz2	T41+T23+T13+T8+T5	322560
submats_8_T41.pbz2	T41+T23+T14+T6+T4	201600
submats_8_T41.pbz2	T41+T23+T14+T7+T4	403200
submats_8_T41.pbz2	T41+T23+T14+T7+T5	604800
submats_8_T41.pbz2	T41+T23+T14+T8+T4	201600
submats_8_T41.pbz2	T41+T23+T14+T8+T5	201600
submats_8_T41.pbz2	T41+T23+T9+T6+T4	40320
submats_8_T42.pbz2	T42+T15+T9+T6+T4	40320
submats_8_T42.pbz2	T42+T16+T10+T6+T4	120960
submats_8_T42.pbz2	T42+T16+T10+T7+T4	120960
submats_8_T42.pbz2	T42+T16+T10+T7+T5	181440
submats_8_T42.pbz2	T42+T16+T9+T6+T4	120960
submats_8_T42.pbz2	T42+T17+T10+T6+T4	161280
submats_8_T42.pbz2	T42+T17+T10+T7+T4	161280
submats_8_T42.pbz2	T42+T17+T10+T7+T5	241920
submats_8_T42.pbz2	T42+T17+T11+T6+T4	80640
submats_8_T42.pbz2	T42+T17+T11+T7+T4	322560
submats_8_T42.pbz2	T42+T17+T11+T7+T5	483840
submats_8_T42.pbz2	T42+T17+T9+T6+T4	80640
submats_8_T42.pbz2	T42+T19+T10+T6+T4	161280
submats_8_T42.pbz2	T42+T19+T10+T7+T4	161280
submats_8_T42.pbz2	T42+T19+T10+T7+T5	241920
submats_8_T42.pbz2	T42+T19+T12+T6+T4	80640
submats_8_T42.pbz2	T42+T19+T12+T7+T4	161280
submats_8_T42.pbz2	T42+T19+T12+T7+T5	241920
submats_8_T42.pbz2	T42+T19+T12+T8+T4	241920
submats_8_T42.pbz2	T42+T19+T12+T8+T5	241920
submats_8_T42.pbz2	T42+T19+T9+T6+T4	80640
submats_8_T42.pbz2	T42+T20+T10+T6+T4	362880
submats_8_T42.pbz2	T42+T20+T10+T7+T4	362880
submats_8_T42.pbz2	T42+T20+T10+T7+T5	544320
submats_8_T42.pbz2	T42+T20+T11+T6+T4	120960
submats_8_T42.pbz2	T42+T20+T11+T7+T4	483840
submats_8_T42.pbz2	T42+T20+T11+T7+T5	725760
submats_8_T42.pbz2	T42+T20+T12+T6+T4	241920
submats_8_T42.pbz2	T42+T20+T12+T7+T4	483840
submats_8_T42.pbz2	T42+T20+T12+T7+T5	725760
submats_8_T42.pbz2	T42+T20+T12+T8+T4	725760
submats_8_T42.pbz2	T42+T20+T12+T8+T5	725760
submats_8_T42.pbz2	T42+T20+T13+T6+T4	362880
submats_8_T42.pbz2	T42+T20+T13+T7+T4	1088640
submats_8_T42.pbz2	T42+T20+T13+T7+T5	1632960

Continued on next page

Table S2 – continued from previous page

File name	Tree sequence	# matrices
submats_8_T42.pbz2	T42+T20+T13+T8+T4	725760
submats_8_T42.pbz2	T42+T20+T13+T8+T5	725760
submats_8_T42.pbz2	T42+T20+T9+T6+T4	120960
submats_8_T42.pbz2	T42+T21+T10+T6+T4	40320
submats_8_T42.pbz2	T42+T21+T10+T7+T4	40320
submats_8_T42.pbz2	T42+T21+T10+T7+T5	60480
submats_8_T42.pbz2	T42+T21+T11+T6+T4	40320
submats_8_T42.pbz2	T42+T21+T11+T7+T4	161280
submats_8_T42.pbz2	T42+T21+T11+T7+T5	241920
submats_8_T42.pbz2	T42+T21+T13+T6+T4	40320
submats_8_T42.pbz2	T42+T21+T13+T7+T4	120960
submats_8_T42.pbz2	T42+T21+T13+T7+T5	181440
submats_8_T42.pbz2	T42+T21+T13+T8+T4	80640
submats_8_T42.pbz2	T42+T21+T13+T8+T5	80640
submats_8_T42.pbz2	T42+T21+T9+T6+T4	40320
submats_8_T42.pbz2	T42+T22+T10+T6+T4	322560
submats_8_T42.pbz2	T42+T22+T10+T7+T4	322560
submats_8_T42.pbz2	T42+T22+T10+T7+T5	483840
submats_8_T42.pbz2	T42+T22+T11+T6+T4	241920
submats_8_T42.pbz2	T42+T22+T11+T7+T4	967680
submats_8_T42.pbz2	T42+T22+T11+T7+T5	1451520
submats_8_T42.pbz2	T42+T22+T12+T6+T4	322560
submats_8_T42.pbz2	T42+T22+T12+T7+T4	645120
submats_8_T42.pbz2	T42+T22+T12+T7+T5	967680
submats_8_T42.pbz2	T42+T22+T12+T8+T4	967680
submats_8_T42.pbz2	T42+T22+T12+T8+T5	967680
submats_8_T42.pbz2	T42+T22+T13+T6+T4	483840
submats_8_T42.pbz2	T42+T22+T13+T7+T4	1451520
submats_8_T42.pbz2	T42+T22+T13+T7+T5	2177280
submats_8_T42.pbz2	T42+T22+T13+T8+T4	967680
submats_8_T42.pbz2	T42+T22+T13+T8+T5	967680
submats_8_T42.pbz2	T42+T22+T9+T6+T4	80640
submats_8_T42.pbz2	T42+T23+T10+T6+T4	161280
submats_8_T42.pbz2	T42+T23+T10+T7+T4	161280
submats_8_T42.pbz2	T42+T23+T10+T7+T5	241920
submats_8_T42.pbz2	T42+T23+T11+T6+T4	80640
submats_8_T42.pbz2	T42+T23+T11+T7+T4	322560
submats_8_T42.pbz2	T42+T23+T11+T7+T5	483840
submats_8_T42.pbz2	T42+T23+T12+T6+T4	80640
submats_8_T42.pbz2	T42+T23+T12+T7+T4	161280
submats_8_T42.pbz2	T42+T23+T12+T7+T5	241920
submats_8_T42.pbz2	T42+T23+T12+T8+T4	241920
submats_8_T42.pbz2	T42+T23+T12+T8+T5	241920

Continued on next page

Table S2 – continued from previous page

File name	Tree sequence	# matrices
submats_8_T42.pbz2	T42+T23+T13+T6+T4	322560
submats_8_T42.pbz2	T42+T23+T13+T7+T4	967680
submats_8_T42.pbz2	T42+T23+T13+T7+T5	1451520
submats_8_T42.pbz2	T42+T23+T13+T8+T4	645120
submats_8_T42.pbz2	T42+T23+T13+T8+T5	645120
submats_8_T42.pbz2	T42+T23+T14+T6+T4	403200
submats_8_T42.pbz2	T42+T23+T14+T7+T4	806400
submats_8_T42.pbz2	T42+T23+T14+T7+T5	1209600
submats_8_T42.pbz2	T42+T23+T14+T8+T4	403200
submats_8_T42.pbz2	T42+T23+T14+T8+T5	403200
submats_8_T42.pbz2	T42+T23+T9+T6+T4	80640
submats_8_T42.pbz2	T42+T24+T10+T6+T4	483840
submats_8_T42.pbz2	T42+T24+T10+T7+T4	483840
submats_8_T42.pbz2	T42+T24+T10+T7+T5	725760
submats_8_T42.pbz2	T42+T24+T11+T6+T4	241920
submats_8_T42.pbz2	T42+T24+T11+T7+T4	967680
submats_8_T42.pbz2	T42+T24+T11+T7+T5	1451520
submats_8_T42.pbz2	T42+T24+T12+T6+T4	322560
submats_8_T42.pbz2	T42+T24+T12+T7+T4	645120
submats_8_T42.pbz2	T42+T24+T12+T7+T5	967680
submats_8_T42.pbz2	T42+T24+T12+T8+T4	967680
submats_8_T42.pbz2	T42+T24+T12+T8+T5	967680
submats_8_T42.pbz2	T42+T24+T13+T6+T4	645120
submats_8_T42.pbz2	T42+T24+T13+T7+T4	1935360
submats_8_T42.pbz2	T42+T24+T13+T7+T5	2903040
submats_8_T42.pbz2	T42+T24+T13+T8+T4	1290240
submats_8_T42.pbz2	T42+T24+T13+T8+T5	1290240
submats_8_T42.pbz2	T42+T24+T14+T6+T4	403200
submats_8_T42.pbz2	T42+T24+T14+T7+T4	806400
submats_8_T42.pbz2	T42+T24+T14+T7+T5	1209600
submats_8_T42.pbz2	T42+T24+T14+T8+T4	403200
submats_8_T42.pbz2	T42+T24+T14+T8+T5	403200
submats_8_T42.pbz2	T42+T24+T9+T6+T4	161280
submats_8_T43.pbz2	T43+T15+T9+T6+T4	20160
submats_8_T43.pbz2	T43+T16+T10+T6+T4	40320
submats_8_T43.pbz2	T43+T16+T10+T7+T4	40320
submats_8_T43.pbz2	T43+T16+T10+T7+T5	60480
submats_8_T43.pbz2	T43+T16+T9+T6+T4	40320
submats_8_T43.pbz2	T43+T17+T10+T6+T4	80640
submats_8_T43.pbz2	T43+T17+T10+T7+T4	80640
submats_8_T43.pbz2	T43+T17+T10+T7+T5	120960
submats_8_T43.pbz2	T43+T17+T11+T6+T4	40320
submats_8_T43.pbz2	T43+T17+T11+T7+T4	161280

Continued on next page

Table S2 – continued from previous page

File name	Tree sequence	# matrices
submats_8_T43.pbz2	T43+T17+T11+T7+T5	241920
submats_8_T43.pbz2	T43+T17+T9+T6+T4	40320
submats_8_T43.pbz2	T43+T19+T10+T6+T4	40320
submats_8_T43.pbz2	T43+T19+T10+T7+T4	40320
submats_8_T43.pbz2	T43+T19+T10+T7+T5	60480
submats_8_T43.pbz2	T43+T19+T12+T6+T4	20160
submats_8_T43.pbz2	T43+T19+T12+T7+T4	40320
submats_8_T43.pbz2	T43+T19+T12+T7+T5	60480
submats_8_T43.pbz2	T43+T19+T12+T8+T4	60480
submats_8_T43.pbz2	T43+T19+T12+T8+T5	60480
submats_8_T43.pbz2	T43+T19+T9+T6+T4	20160
submats_8_T43.pbz2	T43+T20+T10+T6+T4	120960
submats_8_T43.pbz2	T43+T20+T10+T7+T4	120960
submats_8_T43.pbz2	T43+T20+T10+T7+T5	181440
submats_8_T43.pbz2	T43+T20+T11+T6+T4	40320
submats_8_T43.pbz2	T43+T20+T11+T7+T4	161280
submats_8_T43.pbz2	T43+T20+T11+T7+T5	241920
submats_8_T43.pbz2	T43+T20+T12+T6+T4	80640
submats_8_T43.pbz2	T43+T20+T12+T7+T4	161280
submats_8_T43.pbz2	T43+T20+T12+T7+T5	241920
submats_8_T43.pbz2	T43+T20+T12+T8+T4	241920
submats_8_T43.pbz2	T43+T20+T12+T8+T5	241920
submats_8_T43.pbz2	T43+T20+T13+T6+T4	120960
submats_8_T43.pbz2	T43+T20+T13+T7+T4	362880
submats_8_T43.pbz2	T43+T20+T13+T7+T5	544320
submats_8_T43.pbz2	T43+T20+T13+T8+T4	241920
submats_8_T43.pbz2	T43+T20+T13+T8+T5	241920
submats_8_T43.pbz2	T43+T20+T9+T6+T4	40320
submats_8_T43.pbz2	T43+T21+T10+T6+T4	40320
submats_8_T43.pbz2	T43+T21+T10+T7+T4	40320
submats_8_T43.pbz2	T43+T21+T10+T7+T5	60480
submats_8_T43.pbz2	T43+T21+T11+T6+T4	40320
submats_8_T43.pbz2	T43+T21+T11+T7+T4	161280
submats_8_T43.pbz2	T43+T21+T11+T7+T5	241920
submats_8_T43.pbz2	T43+T21+T13+T6+T4	40320
submats_8_T43.pbz2	T43+T21+T13+T7+T4	120960
submats_8_T43.pbz2	T43+T21+T13+T7+T5	181440
submats_8_T43.pbz2	T43+T21+T13+T8+T4	80640
submats_8_T43.pbz2	T43+T21+T13+T8+T5	80640
submats_8_T43.pbz2	T43+T21+T9+T6+T4	40320
submats_8_T43.pbz2	T43+T22+T10+T6+T4	161280
submats_8_T43.pbz2	T43+T22+T10+T7+T4	161280
submats_8_T43.pbz2	T43+T22+T10+T7+T5	241920

Continued on next page

Table S2 – continued from previous page

**File name**	**Tree sequence**	**# matrices**
submats_8_T43.pbz2	T43+T22+T11+T6+T4	120960
submats_8_T43.pbz2	T43+T22+T11+T7+T4	483840
submats_8_T43.pbz2	T43+T22+T11+T7+T5	725760
submats_8_T43.pbz2	T43+T22+T12+T6+T4	161280
submats_8_T43.pbz2	T43+T22+T12+T7+T4	322560
submats_8_T43.pbz2	T43+T22+T12+T7+T5	483840
submats_8_T43.pbz2	T43+T22+T12+T8+T4	483840
submats_8_T43.pbz2	T43+T22+T12+T8+T5	483840
submats_8_T43.pbz2	T43+T22+T13+T6+T4	241920
submats_8_T43.pbz2	T43+T22+T13+T7+T4	725760
submats_8_T43.pbz2	T43+T22+T13+T7+T5	1088640
submats_8_T43.pbz2	T43+T22+T13+T8+T4	483840
submats_8_T43.pbz2	T43+T22+T13+T8+T5	483840
submats_8_T43.pbz2	T43+T22+T9+T6+T4	40320
submats_8_T43.pbz2	T43+T23+T10+T6+T4	80640
submats_8_T43.pbz2	T43+T23+T10+T7+T4	80640
submats_8_T43.pbz2	T43+T23+T10+T7+T5	120960
submats_8_T43.pbz2	T43+T23+T11+T6+T4	40320
submats_8_T43.pbz2	T43+T23+T11+T7+T4	161280
submats_8_T43.pbz2	T43+T23+T11+T7+T5	241920
submats_8_T43.pbz2	T43+T23+T12+T6+T4	40320
submats_8_T43.pbz2	T43+T23+T12+T7+T4	80640
submats_8_T43.pbz2	T43+T23+T12+T7+T5	120960
submats_8_T43.pbz2	T43+T23+T12+T8+T4	120960
submats_8_T43.pbz2	T43+T23+T12+T8+T5	120960
submats_8_T43.pbz2	T43+T23+T13+T6+T4	161280
submats_8_T43.pbz2	T43+T23+T13+T7+T4	483840
submats_8_T43.pbz2	T43+T23+T13+T7+T5	725760
submats_8_T43.pbz2	T43+T23+T13+T8+T4	322560
submats_8_T43.pbz2	T43+T23+T13+T8+T5	322560
submats_8_T43.pbz2	T43+T23+T14+T6+T4	201600
submats_8_T43.pbz2	T43+T23+T14+T7+T4	403200
submats_8_T43.pbz2	T43+T23+T14+T7+T5	604800
submats_8_T43.pbz2	T43+T23+T14+T8+T4	201600
submats_8_T43.pbz2	T43+T23+T14+T8+T5	201600
submats_8_T43.pbz2	T43+T23+T9+T6+T4	40320
submats_8_T43.pbz2	T43+T24+T10+T6+T4	483840
submats_8_T43.pbz2	T43+T24+T10+T7+T4	483840
submats_8_T43.pbz2	T43+T24+T10+T7+T5	725760
submats_8_T43.pbz2	T43+T24+T11+T6+T4	241920
submats_8_T43.pbz2	T43+T24+T11+T7+T4	967680
submats_8_T43.pbz2	T43+T24+T11+T7+T5	1451520
submats_8_T43.pbz2	T43+T24+T12+T6+T4	322560

Continued on next page

Table S2 – continued from previous page

File name	Tree sequence	# matrices
submats_8_T43.pbz2	T43+T24+T12+T7+T4	645120
submats_8_T43.pbz2	T43+T24+T12+T7+T5	967680
submats_8_T43.pbz2	T43+T24+T12+T8+T4	967680
submats_8_T43.pbz2	T43+T24+T12+T8+T5	967680
submats_8_T43.pbz2	T43+T24+T13+T6+T4	645120
submats_8_T43.pbz2	T43+T24+T13+T7+T4	1935360
submats_8_T43.pbz2	T43+T24+T13+T7+T5	2903040
submats_8_T43.pbz2	T43+T24+T13+T8+T4	1290240
submats_8_T43.pbz2	T43+T24+T13+T8+T5	1290240
submats_8_T43.pbz2	T43+T24+T14+T6+T4	403200
submats_8_T43.pbz2	T43+T24+T14+T7+T4	806400
submats_8_T43.pbz2	T43+T24+T14+T7+T5	1209600
submats_8_T43.pbz2	T43+T24+T14+T8+T4	403200
submats_8_T43.pbz2	T43+T24+T14+T8+T5	403200
submats_8_T43.pbz2	T43+T24+T9+T6+T4	161280
submats_8_T43.pbz2	T43+T25+T10+T6+T4	403200
submats_8_T43.pbz2	T43+T25+T10+T7+T4	403200
submats_8_T43.pbz2	T43+T25+T10+T7+T5	604800
submats_8_T43.pbz2	T43+T25+T11+T6+T4	201600
submats_8_T43.pbz2	T43+T25+T11+T7+T4	806400
submats_8_T43.pbz2	T43+T25+T11+T7+T5	1209600
submats_8_T43.pbz2	T43+T25+T12+T6+T4	201600
submats_8_T43.pbz2	T43+T25+T12+T7+T4	403200
submats_8_T43.pbz2	T43+T25+T12+T7+T5	604800
submats_8_T43.pbz2	T43+T25+T12+T8+T4	604800
submats_8_T43.pbz2	T43+T25+T12+T8+T5	604800
submats_8_T43.pbz2	T43+T25+T13+T6+T4	403200
submats_8_T43.pbz2	T43+T25+T13+T7+T4	1209600
submats_8_T43.pbz2	T43+T25+T13+T7+T5	1814400
submats_8_T43.pbz2	T43+T25+T13+T8+T4	806400
submats_8_T43.pbz2	T43+T25+T13+T8+T5	806400
submats_8_T43.pbz2	T43+T25+T14+T6+T4	201600
submats_8_T43.pbz2	T43+T25+T14+T7+T4	403200
submats_8_T43.pbz2	T43+T25+T14+T7+T5	604800
submats_8_T43.pbz2	T43+T25+T14+T8+T4	201600
submats_8_T43.pbz2	T43+T25+T14+T8+T5	201600
submats_8_T43.pbz2	T43+T25+T9+T6+T4	201600
submats_8_T44.pbz2	T44+T15+T9+T6+T4	20160
submats_8_T44.pbz2	T44+T16+T10+T6+T4	80640
submats_8_T44.pbz2	T44+T16+T10+T7+T4	80640
submats_8_T44.pbz2	T44+T16+T10+T7+T5	120960
submats_8_T44.pbz2	T44+T16+T9+T6+T4	80640
submats_8_T44.pbz2	T44+T17+T10+T6+T4	241920

Continued on next page

Table S2 – continued from previous page

File name	Tree sequence	# matrices
submats_8_T44.pbz2	T44+T17+T10+T7+T4	241920
submats_8_T44.pbz2	T44+T17+T10+T7+T5	362880
submats_8_T44.pbz2	T44+T17+T11+T6+T4	120960
submats_8_T44.pbz2	T44+T17+T11+T7+T4	483840
submats_8_T44.pbz2	T44+T17+T11+T7+T5	725760
submats_8_T44.pbz2	T44+T17+T9+T6+T4	120960
submats_8_T44.pbz2	T44+T18+T11+T6+T4	60480
submats_8_T44.pbz2	T44+T18+T11+T7+T4	241920
submats_8_T44.pbz2	T44+T18+T11+T7+T5	362880
submats_8_T44.pbz2	T44+T19+T10+T6+T4	161280
submats_8_T44.pbz2	T44+T19+T10+T7+T4	161280
submats_8_T44.pbz2	T44+T19+T10+T7+T5	241920
submats_8_T44.pbz2	T44+T19+T12+T6+T4	80640
submats_8_T44.pbz2	T44+T19+T12+T7+T4	161280
submats_8_T44.pbz2	T44+T19+T12+T7+T5	241920
submats_8_T44.pbz2	T44+T19+T12+T8+T4	241920
submats_8_T44.pbz2	T44+T19+T12+T8+T5	241920
submats_8_T44.pbz2	T44+T19+T9+T6+T4	80640
submats_8_T44.pbz2	T44+T20+T10+T6+T4	604800
submats_8_T44.pbz2	T44+T20+T10+T7+T4	604800
submats_8_T44.pbz2	T44+T20+T10+T7+T5	907200
submats_8_T44.pbz2	T44+T20+T11+T6+T4	201600
submats_8_T44.pbz2	T44+T20+T11+T7+T4	806400
submats_8_T44.pbz2	T44+T20+T11+T7+T5	1209600
submats_8_T44.pbz2	T44+T20+T12+T6+T4	403200
submats_8_T44.pbz2	T44+T20+T12+T7+T4	806400
submats_8_T44.pbz2	T44+T20+T12+T7+T5	1209600
submats_8_T44.pbz2	T44+T20+T12+T8+T4	1209600
submats_8_T44.pbz2	T44+T20+T12+T8+T5	1209600
submats_8_T44.pbz2	T44+T20+T13+T6+T4	604800
submats_8_T44.pbz2	T44+T20+T13+T7+T4	1814400
submats_8_T44.pbz2	T44+T20+T13+T7+T5	2721600
submats_8_T44.pbz2	T44+T20+T13+T8+T4	1209600
submats_8_T44.pbz2	T44+T20+T13+T8+T5	1209600
submats_8_T44.pbz2	T44+T20+T9+T6+T4	201600
submats_8_T44.pbz2	T44+T21+T10+T6+T4	120960
submats_8_T44.pbz2	T44+T21+T10+T7+T4	120960
submats_8_T44.pbz2	T44+T21+T10+T7+T5	181440
submats_8_T44.pbz2	T44+T21+T11+T6+T4	120960
submats_8_T44.pbz2	T44+T21+T11+T7+T4	483840
submats_8_T44.pbz2	T44+T21+T11+T7+T5	725760
submats_8_T44.pbz2	T44+T21+T13+T6+T4	120960
submats_8_T44.pbz2	T44+T21+T13+T7+T4	362880

Continued on next page

Table S2 – continued from previous page

File name	Tree sequence	# matrices
submats_8_T44.pbz2	T44+T21+T13+T7+T5	544320
submats_8_T44.pbz2	T44+T21+T13+T8+T4	241920
submats_8_T44.pbz2	T44+T21+T13+T8+T5	241920
submats_8_T44.pbz2	T44+T21+T9+T6+T4	120960
submats_8_T44.pbz2	T44+T22+T10+T6+T4	403200
submats_8_T44.pbz2	T44+T22+T10+T7+T4	403200
submats_8_T44.pbz2	T44+T22+T10+T7+T5	604800
submats_8_T44.pbz2	T44+T22+T11+T6+T4	302400
submats_8_T44.pbz2	T44+T22+T11+T7+T4	1209600
submats_8_T44.pbz2	T44+T22+T11+T7+T5	1814400
submats_8_T44.pbz2	T44+T22+T12+T6+T4	403200
submats_8_T44.pbz2	T44+T22+T12+T7+T4	806400
submats_8_T44.pbz2	T44+T22+T12+T7+T5	1209600
submats_8_T44.pbz2	T44+T22+T12+T8+T4	1209600
submats_8_T44.pbz2	T44+T22+T12+T8+T5	1209600
submats_8_T44.pbz2	T44+T22+T13+T6+T4	604800
submats_8_T44.pbz2	T44+T22+T13+T7+T4	1814400
submats_8_T44.pbz2	T44+T22+T13+T7+T5	2721600
submats_8_T44.pbz2	T44+T22+T13+T8+T4	1209600
submats_8_T44.pbz2	T44+T22+T13+T8+T5	1209600
submats_8_T44.pbz2	T44+T22+T9+T6+T4	100800
submats_8_T44.pbz2	T44+T23+T10+T6+T4	483840
submats_8_T44.pbz2	T44+T23+T10+T7+T4	483840
submats_8_T44.pbz2	T44+T23+T10+T7+T5	725760
submats_8_T44.pbz2	T44+T23+T11+T6+T4	241920
submats_8_T44.pbz2	T44+T23+T11+T7+T4	967680
submats_8_T44.pbz2	T44+T23+T11+T7+T5	1451520
submats_8_T44.pbz2	T44+T23+T12+T6+T4	241920
submats_8_T44.pbz2	T44+T23+T12+T7+T4	483840
submats_8_T44.pbz2	T44+T23+T12+T7+T5	725760
submats_8_T44.pbz2	T44+T23+T12+T8+T4	725760
submats_8_T44.pbz2	T44+T23+T12+T8+T5	725760
submats_8_T44.pbz2	T44+T23+T13+T6+T4	967680
submats_8_T44.pbz2	T44+T23+T13+T7+T4	2903040
submats_8_T44.pbz2	T44+T23+T13+T7+T5	4354560
submats_8_T44.pbz2	T44+T23+T13+T8+T4	1935360
submats_8_T44.pbz2	T44+T23+T13+T8+T5	1935360
submats_8_T44.pbz2	T44+T23+T14+T6+T4	1209600
submats_8_T44.pbz2	T44+T23+T14+T7+T4	2419200
submats_8_T44.pbz2	T44+T23+T14+T7+T5	3628800
submats_8_T44.pbz2	T44+T23+T14+T8+T4	1209600
submats_8_T44.pbz2	T44+T23+T14+T8+T5	1209600
submats_8_T44.pbz2	T44+T23+T9+T6+T4	241920

Continued on next page

Table S2 – continued from previous page

File name	Tree sequence	# matrices
submats_8_T44.pbz2	T44+T24+T10+T6+T4	483840
submats_8_T44.pbz2	T44+T24+T10+T7+T4	483840
submats_8_T44.pbz2	T44+T24+T10+T7+T5	725760
submats_8_T44.pbz2	T44+T24+T11+T6+T4	241920
submats_8_T44.pbz2	T44+T24+T11+T7+T4	967680
submats_8_T44.pbz2	T44+T24+T11+T7+T5	1451520
submats_8_T44.pbz2	T44+T24+T12+T6+T4	322560
submats_8_T44.pbz2	T44+T24+T12+T7+T4	645120
submats_8_T44.pbz2	T44+T24+T12+T7+T5	967680
submats_8_T44.pbz2	T44+T24+T12+T8+T4	967680
submats_8_T44.pbz2	T44+T24+T12+T8+T5	967680
submats_8_T44.pbz2	T44+T24+T13+T6+T4	645120
submats_8_T44.pbz2	T44+T24+T13+T7+T4	1935360
submats_8_T44.pbz2	T44+T24+T13+T7+T5	2903040
submats_8_T44.pbz2	T44+T24+T13+T8+T4	1290240
submats_8_T44.pbz2	T44+T24+T13+T8+T5	1290240
submats_8_T44.pbz2	T44+T24+T14+T6+T4	403200
submats_8_T44.pbz2	T44+T24+T14+T7+T4	806400
submats_8_T44.pbz2	T44+T24+T14+T7+T5	1209600
submats_8_T44.pbz2	T44+T24+T14+T8+T4	403200
submats_8_T44.pbz2	T44+T24+T14+T8+T5	403200
submats_8_T44.pbz2	T44+T24+T9+T6+T4	161280
submats_8_T45.pbz2	T45+T15+T9+T6+T4	40320
submats_8_T45.pbz2	T45+T16+T10+T6+T4	40320
submats_8_T45.pbz2	T45+T16+T10+T7+T4	40320
submats_8_T45.pbz2	T45+T16+T10+T7+T5	60480
submats_8_T45.pbz2	T45+T16+T9+T6+T4	40320
submats_8_T45.pbz2	T45+T17+T10+T6+T4	161280
submats_8_T45.pbz2	T45+T17+T10+T7+T4	161280
submats_8_T45.pbz2	T45+T17+T10+T7+T5	241920
submats_8_T45.pbz2	T45+T17+T11+T6+T4	80640
submats_8_T45.pbz2	T45+T17+T11+T7+T4	322560
submats_8_T45.pbz2	T45+T17+T11+T7+T5	483840
submats_8_T45.pbz2	T45+T17+T9+T6+T4	80640
submats_8_T45.pbz2	T45+T18+T11+T6+T4	60480
submats_8_T45.pbz2	T45+T18+T11+T7+T4	241920
submats_8_T45.pbz2	T45+T18+T11+T7+T5	362880
submats_8_T45.pbz2	T45+T20+T10+T6+T4	120960
submats_8_T45.pbz2	T45+T20+T10+T7+T4	120960
submats_8_T45.pbz2	T45+T20+T10+T7+T5	181440
submats_8_T45.pbz2	T45+T20+T11+T6+T4	40320
submats_8_T45.pbz2	T45+T20+T11+T7+T4	161280
submats_8_T45.pbz2	T45+T20+T11+T7+T5	241920

Continued on next page

Table S2 – continued from previous page

File name	Tree sequence	# matrices
submats_8_T45.pbz2	T45+T20+T12+T6+T4	80640
submats_8_T45.pbz2	T45+T20+T12+T7+T4	161280
submats_8_T45.pbz2	T45+T20+T12+T7+T5	241920
submats_8_T45.pbz2	T45+T20+T12+T8+T4	241920
submats_8_T45.pbz2	T45+T20+T12+T8+T5	241920
submats_8_T45.pbz2	T45+T20+T13+T6+T4	120960
submats_8_T45.pbz2	T45+T20+T13+T7+T4	362880
submats_8_T45.pbz2	T45+T20+T13+T7+T5	544320
submats_8_T45.pbz2	T45+T20+T13+T8+T4	241920
submats_8_T45.pbz2	T45+T20+T13+T8+T5	241920
submats_8_T45.pbz2	T45+T20+T9+T6+T4	40320
submats_8_T45.pbz2	T45+T21+T10+T6+T4	40320
submats_8_T45.pbz2	T45+T21+T10+T7+T4	40320
submats_8_T45.pbz2	T45+T21+T10+T7+T5	60480
submats_8_T45.pbz2	T45+T21+T11+T6+T4	40320
submats_8_T45.pbz2	T45+T21+T11+T7+T4	161280
submats_8_T45.pbz2	T45+T21+T11+T7+T5	241920
submats_8_T45.pbz2	T45+T21+T13+T6+T4	40320
submats_8_T45.pbz2	T45+T21+T13+T7+T4	120960
submats_8_T45.pbz2	T45+T21+T13+T7+T5	181440
submats_8_T45.pbz2	T45+T21+T13+T8+T4	80640
submats_8_T45.pbz2	T45+T21+T13+T8+T5	80640
submats_8_T45.pbz2	T45+T21+T9+T6+T4	40320
submats_8_T45.pbz2	T45+T22+T10+T6+T4	161280
submats_8_T45.pbz2	T45+T22+T10+T7+T4	161280
submats_8_T45.pbz2	T45+T22+T10+T7+T5	241920
submats_8_T45.pbz2	T45+T22+T11+T6+T4	120960
submats_8_T45.pbz2	T45+T22+T11+T7+T4	483840
submats_8_T45.pbz2	T45+T22+T11+T7+T5	725760
submats_8_T45.pbz2	T45+T22+T12+T6+T4	161280
submats_8_T45.pbz2	T45+T22+T12+T7+T4	322560
submats_8_T45.pbz2	T45+T22+T12+T7+T5	483840
submats_8_T45.pbz2	T45+T22+T12+T8+T4	483840
submats_8_T45.pbz2	T45+T22+T12+T8+T5	483840
submats_8_T45.pbz2	T45+T22+T13+T6+T4	241920
submats_8_T45.pbz2	T45+T22+T13+T7+T4	725760
submats_8_T45.pbz2	T45+T22+T13+T7+T5	1088640
submats_8_T45.pbz2	T45+T22+T13+T8+T4	483840
submats_8_T45.pbz2	T45+T22+T13+T8+T5	483840
submats_8_T45.pbz2	T45+T22+T9+T6+T4	40320
submats_8_T45.pbz2	T45+T24+T10+T6+T4	120960
submats_8_T45.pbz2	T45+T24+T10+T7+T4	120960
submats_8_T45.pbz2	T45+T24+T10+T7+T5	181440

Continued on next page

Table S2 – continued from previous page

File name	Tree sequence	# matrices
submats_8_T45.pbz2	T45+T24+T11+T6+T4	60480
submats_8_T45.pbz2	T45+T24+T11+T7+T4	241920
submats_8_T45.pbz2	T45+T24+T11+T7+T5	362880
submats_8_T45.pbz2	T45+T24+T12+T6+T4	80640
submats_8_T45.pbz2	T45+T24+T12+T7+T4	161280
submats_8_T45.pbz2	T45+T24+T12+T7+T5	241920
submats_8_T45.pbz2	T45+T24+T12+T8+T4	241920
submats_8_T45.pbz2	T45+T24+T12+T8+T5	241920
submats_8_T45.pbz2	T45+T24+T13+T6+T4	161280
submats_8_T45.pbz2	T45+T24+T13+T7+T4	483840
submats_8_T45.pbz2	T45+T24+T13+T7+T5	725760
submats_8_T45.pbz2	T45+T24+T13+T8+T4	322560
submats_8_T45.pbz2	T45+T24+T13+T8+T5	322560
submats_8_T45.pbz2	T45+T24+T14+T6+T4	100800
submats_8_T45.pbz2	T45+T24+T14+T7+T4	201600
submats_8_T45.pbz2	T45+T24+T14+T7+T5	302400
submats_8_T45.pbz2	T45+T24+T14+T8+T4	100800
submats_8_T45.pbz2	T45+T24+T14+T8+T5	100800
submats_8_T45.pbz2	T45+T24+T9+T6+T4	40320
submats_8_T46.pbz2	T46+T15+T9+T6+T4	40320
submats_8_T46.pbz2	T46+T16+T10+T6+T4	80640
submats_8_T46.pbz2	T46+T16+T10+T7+T4	80640
submats_8_T46.pbz2	T46+T16+T10+T7+T5	120960
submats_8_T46.pbz2	T46+T16+T9+T6+T4	80640
submats_8_T46.pbz2	T46+T17+T10+T6+T4	161280
submats_8_T46.pbz2	T46+T17+T10+T7+T4	161280
submats_8_T46.pbz2	T46+T17+T10+T7+T5	241920
submats_8_T46.pbz2	T46+T17+T11+T6+T4	80640
submats_8_T46.pbz2	T46+T17+T11+T7+T4	322560
submats_8_T46.pbz2	T46+T17+T11+T7+T5	483840
submats_8_T46.pbz2	T46+T17+T9+T6+T4	80640
submats_8_T46.pbz2	T46+T18+T11+T6+T4	60480
submats_8_T46.pbz2	T46+T18+T11+T7+T4	241920
submats_8_T46.pbz2	T46+T18+T11+T7+T5	362880
submats_8_T46.pbz2	T46+T19+T10+T6+T4	80640
submats_8_T46.pbz2	T46+T19+T10+T7+T4	80640
submats_8_T46.pbz2	T46+T19+T10+T7+T5	120960
submats_8_T46.pbz2	T46+T19+T12+T6+T4	40320
submats_8_T46.pbz2	T46+T19+T12+T7+T4	80640
submats_8_T46.pbz2	T46+T19+T12+T7+T5	120960
submats_8_T46.pbz2	T46+T19+T12+T8+T4	120960
submats_8_T46.pbz2	T46+T19+T12+T8+T5	120960
submats_8_T46.pbz2	T46+T19+T9+T6+T4	40320

Continued on next page

Table S2 – continued from previous page

File name	Tree sequence	# matrices
submats_8_T46.pbz2	T46+T20+T10+T6+T4	241920
submats_8_T46.pbz2	T46+T20+T10+T7+T4	241920
submats_8_T46.pbz2	T46+T20+T10+T7+T5	362880
submats_8_T46.pbz2	T46+T20+T11+T6+T4	80640
submats_8_T46.pbz2	T46+T20+T11+T7+T4	322560
submats_8_T46.pbz2	T46+T20+T11+T7+T5	483840
submats_8_T46.pbz2	T46+T20+T12+T6+T4	161280
submats_8_T46.pbz2	T46+T20+T12+T7+T4	322560
submats_8_T46.pbz2	T46+T20+T12+T7+T5	483840
submats_8_T46.pbz2	T46+T20+T12+T8+T4	483840
submats_8_T46.pbz2	T46+T20+T12+T8+T5	483840
submats_8_T46.pbz2	T46+T20+T13+T6+T4	241920
submats_8_T46.pbz2	T46+T20+T13+T7+T4	725760
submats_8_T46.pbz2	T46+T20+T13+T7+T5	1088640
submats_8_T46.pbz2	T46+T20+T13+T8+T4	483840
submats_8_T46.pbz2	T46+T20+T13+T8+T5	483840
submats_8_T46.pbz2	T46+T20+T9+T6+T4	80640
submats_8_T46.pbz2	T46+T21+T10+T6+T4	161280
submats_8_T46.pbz2	T46+T21+T10+T7+T4	161280
submats_8_T46.pbz2	T46+T21+T10+T7+T5	241920
submats_8_T46.pbz2	T46+T21+T11+T6+T4	161280
submats_8_T46.pbz2	T46+T21+T11+T7+T4	645120
submats_8_T46.pbz2	T46+T21+T11+T7+T5	967680
submats_8_T46.pbz2	T46+T21+T13+T6+T4	161280
submats_8_T46.pbz2	T46+T21+T13+T7+T4	483840
submats_8_T46.pbz2	T46+T21+T13+T7+T5	725760
submats_8_T46.pbz2	T46+T21+T13+T8+T4	322560
submats_8_T46.pbz2	T46+T21+T13+T8+T5	322560
submats_8_T46.pbz2	T46+T21+T9+T6+T4	161280
submats_8_T46.pbz2	T46+T22+T10+T6+T4	241920
submats_8_T46.pbz2	T46+T22+T10+T7+T4	241920
submats_8_T46.pbz2	T46+T22+T10+T7+T5	362880
submats_8_T46.pbz2	T46+T22+T11+T6+T4	181440
submats_8_T46.pbz2	T46+T22+T11+T7+T4	725760
submats_8_T46.pbz2	T46+T22+T11+T7+T5	1088640
submats_8_T46.pbz2	T46+T22+T12+T6+T4	241920
submats_8_T46.pbz2	T46+T22+T12+T7+T4	483840
submats_8_T46.pbz2	T46+T22+T12+T7+T5	725760
submats_8_T46.pbz2	T46+T22+T12+T8+T4	725760
submats_8_T46.pbz2	T46+T22+T12+T8+T5	725760
submats_8_T46.pbz2	T46+T22+T13+T6+T4	362880
submats_8_T46.pbz2	T46+T22+T13+T7+T4	1088640
submats_8_T46.pbz2	T46+T22+T13+T7+T5	1632960

Continued on next page

Table S2 – continued from previous page

File name	Tree sequence	# matrices
submats_8_T46.pbz2	T46+T22+T13+T8+T4	725760
submats_8_T46.pbz2	T46+T22+T13+T8+T5	725760
submats_8_T46.pbz2	T46+T22+T9+T6+T4	60480
submats_8_T46.pbz2	T46+T23+T10+T6+T4	322560
submats_8_T46.pbz2	T46+T23+T10+T7+T4	322560
submats_8_T46.pbz2	T46+T23+T10+T7+T5	483840
submats_8_T46.pbz2	T46+T23+T11+T6+T4	161280
submats_8_T46.pbz2	T46+T23+T11+T7+T4	645120
submats_8_T46.pbz2	T46+T23+T11+T7+T5	967680
submats_8_T46.pbz2	T46+T23+T12+T6+T4	161280
submats_8_T46.pbz2	T46+T23+T12+T7+T4	322560
submats_8_T46.pbz2	T46+T23+T12+T7+T5	483840
submats_8_T46.pbz2	T46+T23+T12+T8+T4	483840
submats_8_T46.pbz2	T46+T23+T12+T8+T5	483840
submats_8_T46.pbz2	T46+T23+T13+T6+T4	645120
submats_8_T46.pbz2	T46+T23+T13+T7+T4	1935360
submats_8_T46.pbz2	T46+T23+T13+T7+T5	2903040
submats_8_T46.pbz2	T46+T23+T13+T8+T4	1290240
submats_8_T46.pbz2	T46+T23+T13+T8+T5	1290240
submats_8_T46.pbz2	T46+T23+T14+T6+T4	806400
submats_8_T46.pbz2	T46+T23+T14+T7+T4	1612800
submats_8_T46.pbz2	T46+T23+T14+T7+T5	2419200
submats_8_T46.pbz2	T46+T23+T14+T8+T4	806400
submats_8_T46.pbz2	T46+T23+T14+T8+T5	806400
submats_8_T46.pbz2	T46+T23+T9+T6+T4	161280
submats_8_T46.pbz2	T46+T24+T10+T6+T4	846720
submats_8_T46.pbz2	T46+T24+T10+T7+T4	846720
submats_8_T46.pbz2	T46+T24+T10+T7+T5	1270080
submats_8_T46.pbz2	T46+T24+T11+T6+T4	423360
submats_8_T46.pbz2	T46+T24+T11+T7+T4	1693440
submats_8_T46.pbz2	T46+T24+T11+T7+T5	2540160
submats_8_T46.pbz2	T46+T24+T12+T6+T4	564480
submats_8_T46.pbz2	T46+T24+T12+T7+T4	1128960
submats_8_T46.pbz2	T46+T24+T12+T7+T5	1693440
submats_8_T46.pbz2	T46+T24+T12+T8+T4	1693440
submats_8_T46.pbz2	T46+T24+T12+T8+T5	1693440
submats_8_T46.pbz2	T46+T24+T13+T6+T4	1128960
submats_8_T46.pbz2	T46+T24+T13+T7+T4	3386880
submats_8_T46.pbz2	T46+T24+T13+T7+T5	5080320
submats_8_T46.pbz2	T46+T24+T13+T8+T4	2257920
submats_8_T46.pbz2	T46+T24+T13+T8+T5	2257920
submats_8_T46.pbz2	T46+T24+T14+T6+T4	705600
submats_8_T46.pbz2	T46+T24+T14+T7+T4	1411200

Continued on next page

Table S2 – continued from previous page

File name	Tree sequence	# matrices
submats_8_T46.pbz2	T46+T24+T14+T7+T5	2116800
submats_8_T46.pbz2	T46+T24+T14+T8+T4	705600
submats_8_T46.pbz2	T46+T24+T14+T8+T5	705600
submats_8_T46.pbz2	T46+T24+T9+T6+T4	282240
submats_8_T46.pbz2	T46+T25+T10+T6+T4	604800
submats_8_T46.pbz2	T46+T25+T10+T7+T4	604800
submats_8_T46.pbz2	T46+T25+T10+T7+T5	907200
submats_8_T46.pbz2	T46+T25+T11+T6+T4	302400
submats_8_T46.pbz2	T46+T25+T11+T7+T4	1209600
submats_8_T46.pbz2	T46+T25+T11+T7+T5	1814400
submats_8_T46.pbz2	T46+T25+T12+T6+T4	302400
submats_8_T46.pbz2	T46+T25+T12+T7+T4	604800
submats_8_T46.pbz2	T46+T25+T12+T7+T5	907200
submats_8_T46.pbz2	T46+T25+T12+T8+T4	907200
submats_8_T46.pbz2	T46+T25+T12+T8+T5	907200
submats_8_T46.pbz2	T46+T25+T13+T6+T4	604800
submats_8_T46.pbz2	T46+T25+T13+T7+T4	1814400
submats_8_T46.pbz2	T46+T25+T13+T7+T5	2721600
submats_8_T46.pbz2	T46+T25+T13+T8+T4	1209600
submats_8_T46.pbz2	T46+T25+T13+T8+T5	1209600
submats_8_T46.pbz2	T46+T25+T14+T6+T4	302400
submats_8_T46.pbz2	T46+T25+T14+T7+T4	604800
submats_8_T46.pbz2	T46+T25+T14+T7+T5	907200
submats_8_T46.pbz2	T46+T25+T14+T8+T4	302400
submats_8_T46.pbz2	T46+T25+T14+T8+T5	302400
submats_8_T46.pbz2	T46+T25+T9+T6+T4	302400
submats_8_T47.pbz2	T47+T15+T9+T6+T4	40320
submats_8_T47.pbz2	T47+T16+T10+T6+T4	120960
submats_8_T47.pbz2	T47+T16+T10+T7+T4	120960
submats_8_T47.pbz2	T47+T16+T10+T7+T5	181440
submats_8_T47.pbz2	T47+T16+T9+T6+T4	120960
submats_8_T47.pbz2	T47+T17+T10+T6+T4	241920
submats_8_T47.pbz2	T47+T17+T10+T7+T4	241920
submats_8_T47.pbz2	T47+T17+T10+T7+T5	362880
submats_8_T47.pbz2	T47+T17+T11+T6+T4	120960
submats_8_T47.pbz2	T47+T17+T11+T7+T4	483840
submats_8_T47.pbz2	T47+T17+T11+T7+T5	725760
submats_8_T47.pbz2	T47+T17+T9+T6+T4	120960
submats_8_T47.pbz2	T47+T18+T11+T6+T4	60480
submats_8_T47.pbz2	T47+T18+T11+T7+T4	241920
submats_8_T47.pbz2	T47+T18+T11+T7+T5	362880
submats_8_T47.pbz2	T47+T19+T10+T6+T4	161280
submats_8_T47.pbz2	T47+T19+T10+T7+T4	161280

Continued on next page

Table S2 – continued from previous page

File name	Tree sequence	# matrices
submats_8_T47.pbz2	T47+T19+T10+T7+T5	241920
submats_8_T47.pbz2	T47+T19+T12+T6+T4	80640
submats_8_T47.pbz2	T47+T19+T12+T7+T4	161280
submats_8_T47.pbz2	T47+T19+T12+T7+T5	241920
submats_8_T47.pbz2	T47+T19+T12+T8+T4	241920
submats_8_T47.pbz2	T47+T19+T12+T8+T5	241920
submats_8_T47.pbz2	T47+T19+T9+T6+T4	80640
submats_8_T47.pbz2	T47+T20+T10+T6+T4	483840
submats_8_T47.pbz2	T47+T20+T10+T7+T4	483840
submats_8_T47.pbz2	T47+T20+T10+T7+T5	725760
submats_8_T47.pbz2	T47+T20+T11+T6+T4	161280
submats_8_T47.pbz2	T47+T20+T11+T7+T4	645120
submats_8_T47.pbz2	T47+T20+T11+T7+T5	967680
submats_8_T47.pbz2	T47+T20+T12+T6+T4	322560
submats_8_T47.pbz2	T47+T20+T12+T7+T4	645120
submats_8_T47.pbz2	T47+T20+T12+T7+T5	967680
submats_8_T47.pbz2	T47+T20+T12+T8+T4	967680
submats_8_T47.pbz2	T47+T20+T12+T8+T5	967680
submats_8_T47.pbz2	T47+T20+T13+T6+T4	483840
submats_8_T47.pbz2	T47+T20+T13+T7+T4	1451520
submats_8_T47.pbz2	T47+T20+T13+T7+T5	2177280
submats_8_T47.pbz2	T47+T20+T13+T8+T4	967680
submats_8_T47.pbz2	T47+T20+T13+T8+T5	967680
submats_8_T47.pbz2	T47+T20+T9+T6+T4	161280
submats_8_T47.pbz2	T47+T21+T10+T6+T4	161280
submats_8_T47.pbz2	T47+T21+T10+T7+T4	161280
submats_8_T47.pbz2	T47+T21+T10+T7+T5	241920
submats_8_T47.pbz2	T47+T21+T11+T6+T4	161280
submats_8_T47.pbz2	T47+T21+T11+T7+T4	645120
submats_8_T47.pbz2	T47+T21+T11+T7+T5	967680
submats_8_T47.pbz2	T47+T21+T13+T6+T4	161280
submats_8_T47.pbz2	T47+T21+T13+T7+T4	483840
submats_8_T47.pbz2	T47+T21+T13+T7+T5	725760
submats_8_T47.pbz2	T47+T21+T13+T8+T4	322560
submats_8_T47.pbz2	T47+T21+T13+T8+T5	322560
submats_8_T47.pbz2	T47+T21+T9+T6+T4	161280
submats_8_T47.pbz2	T47+T22+T10+T6+T4	322560
submats_8_T47.pbz2	T47+T22+T10+T7+T4	322560
submats_8_T47.pbz2	T47+T22+T10+T7+T5	483840
submats_8_T47.pbz2	T47+T22+T11+T6+T4	241920
submats_8_T47.pbz2	T47+T22+T11+T7+T4	967680
submats_8_T47.pbz2	T47+T22+T11+T7+T5	1451520
submats_8_T47.pbz2	T47+T22+T12+T6+T4	322560

Continued on next page

Table S2 – continued from previous page

File name	Tree sequence	# matrices
submats_8_T47.pbz2	T47+T22+T12+T7+T4	645120
submats_8_T47.pbz2	T47+T22+T12+T7+T5	967680
submats_8_T47.pbz2	T47+T22+T12+T8+T4	967680
submats_8_T47.pbz2	T47+T22+T12+T8+T5	967680
submats_8_T47.pbz2	T47+T22+T13+T6+T4	483840
submats_8_T47.pbz2	T47+T22+T13+T7+T4	1451520
submats_8_T47.pbz2	T47+T22+T13+T7+T5	2177280
submats_8_T47.pbz2	T47+T22+T13+T8+T4	967680
submats_8_T47.pbz2	T47+T22+T13+T8+T5	967680
submats_8_T47.pbz2	T47+T22+T9+T6+T4	80640
submats_8_T47.pbz2	T47+T23+T10+T6+T4	403200
submats_8_T47.pbz2	T47+T23+T10+T7+T4	403200
submats_8_T47.pbz2	T47+T23+T10+T7+T5	604800
submats_8_T47.pbz2	T47+T23+T11+T6+T4	201600
submats_8_T47.pbz2	T47+T23+T11+T7+T4	806400
submats_8_T47.pbz2	T47+T23+T11+T7+T5	1209600
submats_8_T47.pbz2	T47+T23+T12+T6+T4	201600
submats_8_T47.pbz2	T47+T23+T12+T7+T4	403200
submats_8_T47.pbz2	T47+T23+T12+T7+T5	604800
submats_8_T47.pbz2	T47+T23+T12+T8+T4	604800
submats_8_T47.pbz2	T47+T23+T12+T8+T5	604800
submats_8_T47.pbz2	T47+T23+T13+T6+T4	806400
submats_8_T47.pbz2	T47+T23+T13+T7+T4	2419200
submats_8_T47.pbz2	T47+T23+T13+T7+T5	3628800
submats_8_T47.pbz2	T47+T23+T13+T8+T4	1612800
submats_8_T47.pbz2	T47+T23+T13+T8+T5	1612800
submats_8_T47.pbz2	T47+T23+T14+T6+T4	1008000
submats_8_T47.pbz2	T47+T23+T14+T7+T4	2016000
submats_8_T47.pbz2	T47+T23+T14+T7+T5	3024000
submats_8_T47.pbz2	T47+T23+T14+T8+T4	1008000
submats_8_T47.pbz2	T47+T23+T14+T8+T5	1008000
submats_8_T47.pbz2	T47+T23+T9+T6+T4	201600
submats_8_T47.pbz2	T47+T24+T10+T6+T4	604800
submats_8_T47.pbz2	T47+T24+T10+T7+T4	604800
submats_8_T47.pbz2	T47+T24+T10+T7+T5	907200
submats_8_T47.pbz2	T47+T24+T11+T6+T4	302400
submats_8_T47.pbz2	T47+T24+T11+T7+T4	1209600
submats_8_T47.pbz2	T47+T24+T11+T7+T5	1814400
submats_8_T47.pbz2	T47+T24+T12+T6+T4	403200
submats_8_T47.pbz2	T47+T24+T12+T7+T4	806400
submats_8_T47.pbz2	T47+T24+T12+T7+T5	1209600
submats_8_T47.pbz2	T47+T24+T12+T8+T4	1209600
submats_8_T47.pbz2	T47+T24+T12+T8+T5	1209600

Continued on next page

Table S2 – continued from previous page

File name	Tree sequence	# matrices
submats_8_T47.pbz2	T47+T24+T13+T6+T4	806400
submats_8_T47.pbz2	T47+T24+T13+T7+T4	2419200
submats_8_T47.pbz2	T47+T24+T13+T7+T5	3628800
submats_8_T47.pbz2	T47+T24+T13+T8+T4	1612800
submats_8_T47.pbz2	T47+T24+T13+T8+T5	1612800
submats_8_T47.pbz2	T47+T24+T14+T6+T4	504000
submats_8_T47.pbz2	T47+T24+T14+T7+T4	1008000
submats_8_T47.pbz2	T47+T24+T14+T7+T5	1512000
submats_8_T47.pbz2	T47+T24+T14+T8+T4	504000
submats_8_T47.pbz2	T47+T24+T14+T8+T5	504000
submats_8_T47.pbz2	T47+T24+T9+T6+T4	201600
submats_8_T47.pbz2	T47+T25+T10+T6+T4	241920
submats_8_T47.pbz2	T47+T25+T10+T7+T4	241920
submats_8_T47.pbz2	T47+T25+T10+T7+T5	362880
submats_8_T47.pbz2	T47+T25+T11+T6+T4	120960
submats_8_T47.pbz2	T47+T25+T11+T7+T4	483840
submats_8_T47.pbz2	T47+T25+T11+T7+T5	725760
submats_8_T47.pbz2	T47+T25+T12+T6+T4	120960
submats_8_T47.pbz2	T47+T25+T12+T7+T4	241920
submats_8_T47.pbz2	T47+T25+T12+T7+T5	362880
submats_8_T47.pbz2	T47+T25+T12+T8+T4	362880
submats_8_T47.pbz2	T47+T25+T12+T8+T5	362880
submats_8_T47.pbz2	T47+T25+T13+T6+T4	241920
submats_8_T47.pbz2	T47+T25+T13+T7+T4	725760
submats_8_T47.pbz2	T47+T25+T13+T7+T5	1088640
submats_8_T47.pbz2	T47+T25+T13+T8+T4	483840
submats_8_T47.pbz2	T47+T25+T13+T8+T5	483840
submats_8_T47.pbz2	T47+T25+T14+T6+T4	120960
submats_8_T47.pbz2	T47+T25+T14+T7+T4	241920
submats_8_T47.pbz2	T47+T25+T14+T7+T5	362880
submats_8_T47.pbz2	T47+T25+T14+T8+T4	120960
submats_8_T47.pbz2	T47+T25+T14+T8+T5	120960
submats_8_T47.pbz2	T47+T25+T9+T6+T4	120960
submats_8_T48.pbz2	T48+T15+T9+T6+T4	20160
submats_8_T48.pbz2	T48+T16+T10+T6+T4	40320
submats_8_T48.pbz2	T48+T16+T10+T7+T4	40320
submats_8_T48.pbz2	T48+T16+T10+T7+T5	60480
submats_8_T48.pbz2	T48+T16+T9+T6+T4	40320
submats_8_T48.pbz2	T48+T17+T10+T6+T4	80640
submats_8_T48.pbz2	T48+T17+T10+T7+T4	80640
submats_8_T48.pbz2	T48+T17+T10+T7+T5	120960
submats_8_T48.pbz2	T48+T17+T11+T6+T4	40320
submats_8_T48.pbz2	T48+T17+T11+T7+T4	161280

Continued on next page

Table S2 – continued from previous page

File name	Tree sequence	# matrices
submats_8_T48.pbz2	T48+T17+T11+T7+T5	241920
submats_8_T48.pbz2	T48+T17+T9+T6+T4	40320
submats_8_T48.pbz2	T48+T18+T11+T6+T4	20160
submats_8_T48.pbz2	T48+T18+T11+T7+T4	80640
submats_8_T48.pbz2	T48+T18+T11+T7+T5	120960
submats_8_T48.pbz2	T48+T19+T10+T6+T4	40320
submats_8_T48.pbz2	T48+T19+T10+T7+T4	40320
submats_8_T48.pbz2	T48+T19+T10+T7+T5	60480
submats_8_T48.pbz2	T48+T19+T12+T6+T4	20160
submats_8_T48.pbz2	T48+T19+T12+T7+T4	40320
submats_8_T48.pbz2	T48+T19+T12+T7+T5	60480
submats_8_T48.pbz2	T48+T19+T12+T8+T4	60480
submats_8_T48.pbz2	T48+T19+T12+T8+T5	60480
submats_8_T48.pbz2	T48+T19+T9+T6+T4	20160
submats_8_T48.pbz2	T48+T20+T10+T6+T4	120960
submats_8_T48.pbz2	T48+T20+T10+T7+T4	120960
submats_8_T48.pbz2	T48+T20+T10+T7+T5	181440
submats_8_T48.pbz2	T48+T20+T11+T6+T4	40320
submats_8_T48.pbz2	T48+T20+T11+T7+T4	161280
submats_8_T48.pbz2	T48+T20+T11+T7+T5	241920
submats_8_T48.pbz2	T48+T20+T12+T6+T4	80640
submats_8_T48.pbz2	T48+T20+T12+T7+T4	161280
submats_8_T48.pbz2	T48+T20+T12+T7+T5	241920
submats_8_T48.pbz2	T48+T20+T12+T8+T4	241920
submats_8_T48.pbz2	T48+T20+T12+T8+T5	241920
submats_8_T48.pbz2	T48+T20+T13+T6+T4	120960
submats_8_T48.pbz2	T48+T20+T13+T7+T4	362880
submats_8_T48.pbz2	T48+T20+T13+T7+T5	544320
submats_8_T48.pbz2	T48+T20+T13+T8+T4	241920
submats_8_T48.pbz2	T48+T20+T13+T8+T5	241920
submats_8_T48.pbz2	T48+T20+T9+T6+T4	40320
submats_8_T48.pbz2	T48+T21+T10+T6+T4	40320
submats_8_T48.pbz2	T48+T21+T10+T7+T4	40320
submats_8_T48.pbz2	T48+T21+T10+T7+T5	60480
submats_8_T48.pbz2	T48+T21+T11+T6+T4	40320
submats_8_T48.pbz2	T48+T21+T11+T7+T4	161280
submats_8_T48.pbz2	T48+T21+T11+T7+T5	241920
submats_8_T48.pbz2	T48+T21+T13+T6+T4	40320
submats_8_T48.pbz2	T48+T21+T13+T7+T4	120960
submats_8_T48.pbz2	T48+T21+T13+T7+T5	181440
submats_8_T48.pbz2	T48+T21+T13+T8+T4	80640
submats_8_T48.pbz2	T48+T21+T13+T8+T5	80640
submats_8_T48.pbz2	T48+T21+T9+T6+T4	40320

Continued on next page

Table S2 – continued from previous page

File name	Tree sequence	# matrices
submats_8_T48.pbz2	T48+T22+T10+T6+T4	80640
submats_8_T48.pbz2	T48+T22+T10+T7+T4	80640
submats_8_T48.pbz2	T48+T22+T10+T7+T5	120960
submats_8_T48.pbz2	T48+T22+T11+T6+T4	60480
submats_8_T48.pbz2	T48+T22+T11+T7+T4	241920
submats_8_T48.pbz2	T48+T22+T11+T7+T5	362880
submats_8_T48.pbz2	T48+T22+T12+T6+T4	80640
submats_8_T48.pbz2	T48+T22+T12+T7+T4	161280
submats_8_T48.pbz2	T48+T22+T12+T7+T5	241920
submats_8_T48.pbz2	T48+T22+T12+T8+T4	241920
submats_8_T48.pbz2	T48+T22+T12+T8+T5	241920
submats_8_T48.pbz2	T48+T22+T13+T6+T4	120960
submats_8_T48.pbz2	T48+T22+T13+T7+T4	362880
submats_8_T48.pbz2	T48+T22+T13+T7+T5	544320
submats_8_T48.pbz2	T48+T22+T13+T8+T4	241920
submats_8_T48.pbz2	T48+T22+T13+T8+T5	241920
submats_8_T48.pbz2	T48+T22+T9+T6+T4	20160
submats_8_T48.pbz2	T48+T23+T10+T6+T4	80640
submats_8_T48.pbz2	T48+T23+T10+T7+T4	80640
submats_8_T48.pbz2	T48+T23+T10+T7+T5	120960
submats_8_T48.pbz2	T48+T23+T11+T6+T4	40320
submats_8_T48.pbz2	T48+T23+T11+T7+T4	161280
submats_8_T48.pbz2	T48+T23+T11+T7+T5	241920
submats_8_T48.pbz2	T48+T23+T12+T6+T4	40320
submats_8_T48.pbz2	T48+T23+T12+T7+T4	80640
submats_8_T48.pbz2	T48+T23+T12+T7+T5	120960
submats_8_T48.pbz2	T48+T23+T12+T8+T4	120960
submats_8_T48.pbz2	T48+T23+T12+T8+T5	120960
submats_8_T48.pbz2	T48+T23+T13+T6+T4	161280
submats_8_T48.pbz2	T48+T23+T13+T7+T4	483840
submats_8_T48.pbz2	T48+T23+T13+T7+T5	725760
submats_8_T48.pbz2	T48+T23+T13+T8+T4	322560
submats_8_T48.pbz2	T48+T23+T13+T8+T5	322560
submats_8_T48.pbz2	T48+T23+T14+T6+T4	201600
submats_8_T48.pbz2	T48+T23+T14+T7+T4	403200
submats_8_T48.pbz2	T48+T23+T14+T7+T5	604800
submats_8_T48.pbz2	T48+T23+T14+T8+T4	201600
submats_8_T48.pbz2	T48+T23+T14+T8+T5	201600
submats_8_T48.pbz2	T48+T23+T9+T6+T4	40320
submats_8_T48.pbz2	T48+T24+T10+T6+T4	120960
submats_8_T48.pbz2	T48+T24+T10+T7+T4	120960
submats_8_T48.pbz2	T48+T24+T10+T7+T5	181440
submats_8_T48.pbz2	T48+T24+T11+T6+T4	60480

Continued on next page

Table S2 – continued from previous page

File name	Tree sequence	# matrices
submats_8_T48.pbz2	T48+T24+T11+T7+T4	241920
submats_8_T48.pbz2	T48+T24+T11+T7+T5	362880
submats_8_T48.pbz2	T48+T24+T12+T6+T4	80640
submats_8_T48.pbz2	T48+T24+T12+T7+T4	161280
submats_8_T48.pbz2	T48+T24+T12+T7+T5	241920
submats_8_T48.pbz2	T48+T24+T12+T8+T4	241920
submats_8_T48.pbz2	T48+T24+T12+T8+T5	241920
submats_8_T48.pbz2	T48+T24+T13+T6+T4	161280
submats_8_T48.pbz2	T48+T24+T13+T7+T4	483840
submats_8_T48.pbz2	T48+T24+T13+T7+T5	725760
submats_8_T48.pbz2	T48+T24+T13+T8+T4	322560
submats_8_T48.pbz2	T48+T24+T13+T8+T5	322560
submats_8_T48.pbz2	T48+T24+T14+T6+T4	100800
submats_8_T48.pbz2	T48+T24+T14+T7+T4	201600
submats_8_T48.pbz2	T48+T24+T14+T7+T5	302400
submats_8_T48.pbz2	T48+T24+T14+T8+T4	100800
submats_8_T48.pbz2	T48+T24+T14+T8+T5	100800
submats_8_T48.pbz2	T48+T24+T9+T6+T4	40320
submats_8_T48.pbz2	T48+T25+T10+T6+T4	40320
submats_8_T48.pbz2	T48+T25+T10+T7+T4	40320
submats_8_T48.pbz2	T48+T25+T10+T7+T5	60480
submats_8_T48.pbz2	T48+T25+T11+T6+T4	20160
submats_8_T48.pbz2	T48+T25+T11+T7+T4	80640
submats_8_T48.pbz2	T48+T25+T11+T7+T5	120960
submats_8_T48.pbz2	T48+T25+T12+T6+T4	20160
submats_8_T48.pbz2	T48+T25+T12+T7+T4	40320
submats_8_T48.pbz2	T48+T25+T12+T7+T5	60480
submats_8_T48.pbz2	T48+T25+T12+T8+T4	60480
submats_8_T48.pbz2	T48+T25+T12+T8+T5	60480
submats_8_T48.pbz2	T48+T25+T13+T6+T4	40320
submats_8_T48.pbz2	T48+T25+T13+T7+T4	120960
submats_8_T48.pbz2	T48+T25+T13+T7+T5	181440
submats_8_T48.pbz2	T48+T25+T13+T8+T4	80640
submats_8_T48.pbz2	T48+T25+T13+T8+T5	80640
submats_8_T48.pbz2	T48+T25+T14+T6+T4	20160
submats_8_T48.pbz2	T48+T25+T14+T7+T4	40320
submats_8_T48.pbz2	T48+T25+T14+T7+T5	60480
submats_8_T48.pbz2	T48+T25+T14+T8+T4	20160
submats_8_T48.pbz2	T48+T25+T14+T8+T5	20160
submats_8_T48.pbz2	T48+T25+T9+T6+T4	20160

2 Synthetic data and regular vines with smallest AIC after fitting all regular vines included in Chimera

2.1 Regular vines on 4 nodes

2.1.1 Regular vine used to generate synthetic data

Treeequivalent class: T4
File: submats_4_T4.pbz2
Index of matrix in file: 12

$$M_1 = \begin{bmatrix} 1 & 3 & 3 & 3 \\ 3 & 2 & 2 & 0 \\ 2 & 1 & 0 & 0 \\ 4 & 0 & 0 & 0 \end{bmatrix}$$

Tree 1	Gumbel	Gaussian	Gumbel
Tree 2	Gaussian	Clayton	
Tree 3	Clayton		

Table S3: Bivariate copulas corresponding to the trees on each level of the regular vine corresponding to M_1

Tree 1	4.5	0.8	4.5
Tree 2	0.8	4.5	
Tree 3	4.5		

Table S4: Parameters for the bivariate copulas in Table S3

2.1.2 Regular vine on 4 elements with smallest AIC after fitting all regular vines on 4 nodes.

Total calculation time [h]	0.0
AIC score of R_1	-10017
Tree equivalence class of R_1	T4

Table S5: General characteristics of brute-force fitting regular vines on 4 nodes

$$R_1 = \begin{bmatrix} 1 & 3 & 3 & 3 \\ 3 & 2 & 2 & 0 \\ 2 & 1 & 0 & 0 \\ 4 & 0 & 0 & 0 \end{bmatrix}$$

Tree 1	Gumbel	Gaussian	Gumbel
Tree 2	Gaussian	Clayton	
Tree 3	Clayton		

Table S6: Bivariate copulas corresponding to the trees on each level of the regular vine corresponding to R_1

Tree 1	4.54	0.81	4.61
Tree 2	0.8	4.37	
Tree 3	4.55		

Table S7: Parameters for the bivariate copulas in Table S6

2.2 Regular vines on 5 nodes

2.2.1 Regular vine used to generate synthetic

Tree-equivalent class: T7+T4
File: submats_5_T7.pbz2
Index of matrix in file: 144

$$M_2 = \begin{bmatrix} 2 & 3 & 3 & 5 & 5 \\ 3 & 4 & 5 & 3 & 0 \\ 4 & 5 & 4 & 0 & 0 \\ 5 & 2 & 0 & 0 & 0 \\ 1 & 0 & 0 & 0 & 0 \end{bmatrix}$$

Tree 1	Gumbel	Gaussian	Gumbel	Clayton
Tree 2	Gaussian	Clayton	Gumbel	
Tree 3	Clayton	Gumbel		
Tree 4	Gumbel			

Table S8: Bivariate copulas corresponding to the trees on each level of the regular vine represented by M_2

Tree 1	4.5	0.8	4.5	4.5
Tree 2	0.8	4.5	4.5	
Tree 3	4.5	4.5		
Tree 4	4.5			

Table S9: Parameters for the bivariate copulas in Table S8

2.2.2 Regular vine on 5 elements with smallest AIC after fitting all regular vines on 5 nodes.

Total calculation time [h]	0.03
AIC score	-17518
Tree equivalence class of R_2	T7+T4

Table S10: General characteristics of brute-force fitting regular vines on 5 nodes

$$R_2 = \begin{bmatrix} 2 & 3 & 3 & 5 & 5 \\ 3 & 4 & 5 & 3 & 0 \\ 4 & 5 & 4 & 0 & 0 \\ 5 & 2 & 0 & 0 & 0 \\ 1 & 0 & 0 & 0 & 0 \end{bmatrix}$$

Tree 1	Gumbel	Gaussian	Gumbel	Clayton
Tree 2	Gaussian	Joe 180°	Gumbel	
Tree 3	Clayton	Gumbel		
Tree 4	Gaussian			

Table S11: Bivariate copulas corresponding to the trees on each level of the regular vine corresponding to R_2

Tree 1	4.39	0.8	4.53	4.67
Tree 2	0.81	5.33	4.51	
Tree 3	4.39	4.02		
Tree 4	0.9			

Table S12: Parameters for the bivariate copulas in Table S11

2.3 Regular vines on 6 nodes

2.3.1 Regular vine used to generate synthetic

Vine Type: T11+T7+T4
File: submats_6_T11.pbz2
Index of matrix in file: 1112

$$M_3 = \begin{bmatrix} 6 & 4 & 3 & 3 & 6 & 6 \\ 3 & 3 & 6 & 6 & 3 & 0 \\ 2 & 6 & 4 & 4 & 0 & 0 \\ 4 & 2 & 2 & 0 & 0 & 0 \\ 1 & 1 & 0 & 0 & 0 & 0 \\ 5 & 0 & 0 & 0 & 0 & 0 \end{bmatrix}$$

Tree 1	Gumbel	Gaussian	Gumbel	Clayton	Gaussian
Tree 2	Gaussian	Clayton	Gumbel	Gaussian	
Tree 3	Clayton	Gumbel	Gumbel		
Tree 4	Gumbel	Clayton			
Tree 5	Gumbel				

Table S13: Bivariate copulas corresponding to the trees on each level of the regular vine corresponding to M_3

Tree 1	4.5	0.8	4.5	4.5	0.8
Tree 2	0.8	4.5	4.5	0.8	
Tree 3	4.5	4.5	4.5		
Tree 4	4.5	4.5			
Tree 5	4.5				

Table S14: Parameters for the bivariate copulas in Table S13

2.3.2 Regular vine on 6 elements with smallest AIC after fitting all regular vines on 6 nodes.

Total calculation time [h]	1.84
AIC score	-23764
Tree equivalence class of R_3	T11+T7+T4

Table S15: General characteristics of brute-force fitting regular vines on 5 nodes

$$R_3 = \begin{bmatrix} 6 & 4 & 3 & 3 & 6 & 6 \\ 3 & 3 & 6 & 6 & 3 & 0 \\ 2 & 6 & 4 & 4 & 0 & 0 \\ 4 & 2 & 2 & 0 & 0 & 0 \\ 1 & 1 & 0 & 0 & 0 & 0 \\ 5 & 0 & 0 & 0 & 0 & 0 \end{bmatrix}$$

Tree 1	Gumbel	Gaussian	Gumbel	Joe 180°	Gaussian
Tree 2	Gaussian	Clayton	Gumbel	Gaussian	
Tree 3	Joe 180°	Gumbel	Gumbel		
Tree 4	Gumbel	Joe 180°			
Tree 5	Gumbel				

Table S16: Bivariate copulas corresponding to the trees on each level of the regular vine corresponding to R_3

Tree 1	4.37	0.8	4.57	5.24	0.8
Tree 2	0.81	4.54	4.41	0.8	
Tree 3	5.13	4.33	4.27		
Tree 4	3.91	3.97			
Tree 5	1.51				

Table S17: Parameters for the bivariate copulas in Table S16

2.4 Regular vines on 7 nodes

2.4.1 Regular vine used to generate synthetic

Vine Type: T23+T13+T8+T5
File: submats_7_T23.pbz2
Index of matrix in file: 486866

$$M_4 = \begin{bmatrix} 1 & 2 & 2 & 2 & 1 & 4 & 4 \\ 2 & 3 & 1 & 1 & 4 & 1 & 0 \\ 3 & 1 & 3 & 4 & 2 & 0 & 0 \\ 5 & 5 & 4 & 3 & 0 & 0 & 0 \\ 7 & 4 & 5 & 0 & 0 & 0 & 0 \\ 4 & 7 & 0 & 0 & 0 & 0 & 0 \\ 6 & 0 & 0 & 0 & 0 & 0 & 0 \end{bmatrix}$$

Tree 1	Gumbel	Gaussian	Gumbel	Clayton	Gaussian	Clayton
Tree 2	Gaussian	Clayton	Gumbel	Gaussian	Gumbel	
Tree 3	Clayton	Gumbel	Gumbel	Gaussian		
Tree 4	Gumbel	Clayton	Clayton			
Tree 5	Gumbel	Gumbel				
Tree 6	Clayton					

Table S18: Bivariate copulas corresponding to the trees on each level of the regular vine corresponding to M_4

Tree 1	4.5	0.8	4.5	4.5	0.8	4.5
Tree 2	0.8	4.5	4.5	0.8	4.5	
Tree 3	4.5	4.5	4.5	0.8		
Tree 4	4.5	4.5	4.5			
Tree 5	4.5	4.5				
Tree 6	4.5					

Table S19: Parameters for the bivariate copulas in Table S18

2.4.2 Regular vine on 7 elements with smallest AIC after fitting all regular vines on 7 nodes.

Total calculation time [h]	292.36
AIC score	-34340
Tree equivalence class of R_4	T23+T13+T8+T4

Table S20: General characteristics of brute-force fitting regular vines on 7 nodes

$$R_4 = \begin{bmatrix} 1 & 2 & 2 & 2 & 1 & 4 & 4 \\ 2 & 1 & 3 & 1 & 4 & 1 & 0 \\ 3 & 3 & 1 & 4 & 2 & 0 & 0 \\ 5 & 4 & 4 & 3 & 0 & 0 & 0 \\ 4 & 7 & 7 & 0 & 0 & 0 & 0 \\ 7 & 5 & 0 & 0 & 0 & 0 & 0 \\ 6 & 0 & 0 & 0 & 0 & 0 & 0 \end{bmatrix}$$

Tree 1	Gumbel	Gumbel	Gaussian	Clayton	Gaussian	Clayton
Tree 2	Gaussian	Gumbel	Clayton	Gaussian	Gumbel	
Tree 3	Clayton	Gumbel	Gumbel	Gaussian		
Tree 4	Gumbel	Clayton	Gaussian			
Tree 5	Gumbel	Clayton 180°				
Tree 6	Gumbel 180°					

Table S21: Bivariate copulas corresponding to the trees on each level of the regular vine corresponding to R_4

Tree 1	4.52	4.52	0.8	4.42	0.8	4.41
Tree 2	0.8	4.41	4.37	0.8	4.51	
Tree 3	4.71	4.73	4.63	0.81		
Tree 4	3.9	3.76	0.97			
Tree 5	3.89	0.24				
Tree 6	1.63					

Table S22: Parameters for the bivariate copulas in Table S21

2.5 Regular vines on 8 nodes

2.5.1 Regular vine used to generate synthetic

Tree: T31+T20+T10+T6+T4

$$M_5 = \begin{bmatrix} 7 & 7 & 1 & 8 & 6 & 8 & 7 & 7 \\ 8 & 8 & 8 & 6 & 8 & 7 & 8 & 0 \\ 6 & 6 & 6 & 4 & 7 & 6 & 0 & 0 \\ 5 & 4 & 4 & 7 & 4 & 0 & 0 & 0 \\ 4 & 1 & 7 & 1 & 0 & 0 & 0 & 0 \\ 1 & 2 & 2 & 0 & 0 & 0 & 0 & 0 \\ 2 & 5 & 0 & 0 & 0 & 0 & 0 & 0 \\ 3 & 0 & 0 & 0 & 0 & 0 & 0 & 0 \end{bmatrix}$$

Tree 1	Gumbel	Gaussian	Gaussian	Clayton	Gumbel	Clayton	Clayton
Tree 2	Gumbel	Clayton	Gumbel	Clayton	Gumbel	Clayton	
Tree 3	Gaussian	Clayton	Gumbel	Gaussian	Gaussian		
Tree 4	Clayton	Gumbel	Gaussian	Clayton			
Tree 5	Gumbel	Clayton	Gaussian				
Tree 6	Gumbel	Clayton					
Tree 7	Clayton						

Table S23: Bivariate copulas corresponding to the trees on each level of the regular vine corresponding to M_5

Tree 1	4.5	0.8	0.8	4.5	4.5	4.5	4.5
Tree 2	4.5	4.5	4.5	4.5	4.5	4.5	
Tree 3	0.8	4.5	4.5	0.8	0.8		
Tree 4	4.5	4.5	0.8	4.5			
Tree 5	4.5	4.5	0.8				
Tree 6	4.5	4.5					
Tree 7	4.5						

Table S24: Parameters for the bivariate copulas in Table S23

2.5.2 Regular vine on 8 elements with smallest AIC after fitting all regular vines on 8 nodes.

Total calculation time [h]	106442.03
AIC score	-38480
Tree equivalence class of R_5	T31+T20+T10+T6+T4

Table S25: General characteristics of brute-force fitting regular vines on 8 nodes

$$R_5 = \begin{bmatrix} 7 & 7 & 1 & 8 & 6 & 8 & 7 & 7 \\ 8 & 8 & 8 & 6 & 8 & 7 & 8 & 0 \\ 6 & 6 & 6 & 4 & 7 & 6 & 0 & 0 \\ 5 & 4 & 4 & 7 & 4 & 0 & 0 & 0 \\ 4 & 1 & 7 & 1 & 0 & 0 & 0 & 0 \\ 1 & 2 & 2 & 0 & 0 & 0 & 0 & 0 \\ 2 & 5 & 0 & 0 & 0 & 0 & 0 & 0 \\ 3 & 0 & 0 & 0 & 0 & 0 & 0 & 0 \end{bmatrix}$$

Tree 1	Gumbel	Gaussian	Gaussian	Clayton	Gumbel	Clayton	Clayton
Tree 2	Gumbel	Clayton	Gumbel	Clayton	Gumbel	Clayton	
Tree 3	Gaussian	Clayton	Gumbel	Gaussian	Gaussian		
Tree 4	Gumbel 180°	Gumbel	Gaussian	Clayton			
Tree 5	Frank	Gumbel 180°	Gaussian				
Tree 6	Joe 270°	Frank					
Tree 7	Joe 270°						

Table S26: Bivariate copulas corresponding to the trees on each level of the regular vine corresponding to R_5

Tree 1	4.36	0.79	0.79	4.5	4.6	4.68	4.49
Tree 2	4.62	4.72	4.57	4.3	4.28	4.26	
Tree 3	0.8	4.2	4.51	0.79	0.8		
Tree 4	2.74	2.84	0.75	4.87			
Tree 5	8.01	1.62	0.75				
Tree 6	1.16	4.46					
Tree 7	1.15						

Table S27: Parameters for the bivariate copulas in Table S26

3 Brute-force computational process to find the best fit for 4, 5, 6, 7 and 8 nodes using Chimera

3.1 Introduction

In this supplement we briefly describe the brute-force process used to fit regular vines on 4, 5, 6, 7 and 8 variables to data using Chimera. All possible regular vine matrices included in Chimera have been fitted to data and Akaike's Information Criterion (AIC) has been used to select the "best" vine. A high performance computer has been used [3] in order to perform calculations.

3.2 Parallel processing

In the early days of computing a task conducted on the computer was specified as series of commands executed one after each other. If multiple loops needed to be executed, a traditional loop was set up in order to run the same kind of commands multiple times. Over the years multi-core processors were developed and were able to process independent commands in parallel. The effect of this was that the computing time reduced with the application of parallel computing. In past years *high performance clusters* are created with multiple nodes with multiple cores. On these clusters, users can supply *tasks* to the cluster which will be conducted in parallel on the nodes available in the cluster.

The benefit in performance is strongly related to how the total number of tasks is split up in to multiple parallel task. A large task split up into 2 or 10 smaller ones will result roughly into an elapsed times (required time to run the full analysis) of half or 10% of the regular sequential analysis. Although initialising a parallel computing task, as well as post-processing the results will require still effort, the application of parallel computing will result in significant reduction of elapsed time.

The Delft High Performance Computing Cluster (DHPC) [3] is a system which contains more than 400 nodes with 48 cores each, offering more than 20000 cores for parallel processing. The operating system of the DHPC is Red Hat Enterprise Linux 8 [1]. To provide access to the resources the *Slurm* job resource manager produced by SchedMD [2] is used. In general, *Slurm* takes care of distributing queued tasks to the different nodes.

3.3 General approach

The computational job discussed in this paper consists on running over all possible regular vines to find the best suitable vine for a given data set according to the minimum AIC. Each different regular vine matrix included in Chimera is tested. The authors restricted in this process to fit only one-parameter copulas.

The used module is pyvinelopulib [8] which is a Python [7] interface to Vinecopulib. Vineopulib is a header-only C++ library for vine copula models based on Eigen [4]. The total set of matrices to be tested is split up into different files according to their tree-equivalent class. If these different files

contained more than 20000 different matrices, these files were further split into jobs containing 20000 matrices at most.

In Table S28 the number of files are presented. The initial split-up is based on the different non-isomorphic tree types (see Table 1 of Supplement 1). For example, on 4 nodes there are 2 non-isomorphic trees: T4 and T5. For 5 nodes, T6, T7 and T8 and so on. The second split-up is that files are split according to trees in the lower levels of the regular vine. For 4 nodes (as explained in supplement 1) there are no trees after T4 or T5. For 5 nodes the following tree-equivalent classes exist: T6+T4, T7+T4, T7+T5, T8+T4 and T8+T5. A similar procedure is followed for regular vines on 6, 7 and 8 nodes. In case the number of matrices for a particular tree-equivalent class of regular vine is larger than 20000, the specific job was split-up into parts of 20000 resulting in an equivalent number of files. Tree-equivalent classes of regular vines with more than 20000 matrices occur on 7 and 8 nodes in Chimera.

The configuration of a single job is based on the input of a file containing all matrices of a tree-equivalent class and the interval of the matrices to be processed (i.e 20000 matrices at most) within the job. For all jobs the data is saved and processed afterwards, to find the best suitable matrix and copulas to describe the data as presented in Supplement 2.

nodes	non-isomorphic trees	tree-equivalent classes	# of files
4	2	2	2
5	3	5	5
6	6	22	22
7	11	136	147
8	23	1464	34259

Table S28: Number of files used to fit all regular vines included in Chimera to synthetic data

Theoretically, the best way to divide tasks in parallel would be to split jobs with files containing only 1 regular vine matrix. However, in that case a penalty on post-processing of the results would rise. The results were saved in ASCII files. After finalising the calculations, the results need to be combined to find the best regular vine fitting the synthetic data according to minimum AIC. The authors arbitrarily choose for 20000 as a practical amount of matrices in one sub-file.

3.4 Calculations

The High Performance Cluster is used by multiple researchers simultaneously. Theoretically, the total amount of jobs specifying 20000 matrices could be pushed to *Slurm* at once. Pushing almost 35000 jobs would overload the queue. For this reason, the authors supplied the jobs in batches of 2000 to 4000 jobs at a time. An average job of 20000 matrices used 2GB to 4GB of RAM, which

Nodes	Calculation time	Elapsed time (indicative)
4	0:00	0:00
5	0:02	0:00
6	1:51	0:10
7	292:22	3:20
8	106442:02	75:00

Table S29: Calculation times for fitting all regular vines included in Chimera to synthetic data

depends on the size of the file. A job ran for about 3:10 to 3:30 hours, so within 4 to 5 hours, a total batch of 2000 to 4000 jobs would have ran, depending on the occupation of the High Performance Cluster. After finalising a batch, a new batch was pushed to the queue until all jobs would have ran. In the jobs, the elapsed time of the job was logged and the total calculation time can be found by adding all the elapsed times to each other. Effectively, the total time of calculation for 8 nodes is equivalent to 11.7 years, which is reduced to 3 days and a few hours thanks to the use of the HPC and parallel computing (see table S29). The elapsed times are indicative because these are not logged during the process. In order to decrease the elapsed time, one could split the jobs into even smaller pieces or pushing more jobs in one batch. This process depends on the capacity of the High Performance Cluster as well as its occupancy. Next to the capacity, the pyvinecopulib is still under development and could be possibly improved.

References

[1] Red hat corporation, https://www.redhat.com/en.

[2] Schedmd - slurm workload manager software, https://www.schedmd.com/.

[3] Delft High Performance Computing Centre (DHPC). DelftBlue Supercomputer (Phase 1). `https://www.tudelft.nl/dhpc/ark:/44463/DelftBluePhase1`, 2022.

[4] Gaël Guennebaud, Benoît Jacob, et al. Eigen v3. http://eigen.tuxfamily.org, 2010.

[5] Oswaldo Morales-Nápoles. Bayesian belief nets and vines in aviation safety and other applications. PhD Thesis, Delft Institute of Applied Mathematics, TU Delft, 2010.

[6] Oswaldo Morales-Nápoles. Counting vines. In *Dependence Modeling: Vine Copula Handbook*, page 189 – 218, 2010.

[7] Python Core Team. *Python: A dynamic, open source programming language.* Python Software Foundation, 2019. Python version 3.7.

[8] Thibault Vatter and Thomas Nagler. Pyvinecopulib 0.6.1 - https://vinecopulib.github.io/pyvinecopulib/, 2022.
